# All-trans retinoic acid protects piglets from TGEV-induced diarrhea and intestinal epithelial apoptosis by modulating redox status and endoplasmic reticulum stress pathways

**DOI:** 10.1093/jas/skaf356

**Published:** 2025-10-25

**Authors:** Junning Pu, Haihui Liu, Xinge Li, Daiwen Chen, Gang Tian, Jun He, Ping Zheng, Hui Yan, Aimin Wu, Xiangbing Mao, Junqiu Luo, Bing Yu

**Affiliations:** Animal Nutrition Institute, Animal Nutrition and Efficient Feed Utilization Key Laboratory of Sichuan Province, Sichuan Agricultural University, Chengdu 611130, China; Animal Nutrition Institute, Animal Nutrition and Efficient Feed Utilization Key Laboratory of Sichuan Province, Sichuan Agricultural University, Chengdu 611130, China; Animal Nutrition Institute, Animal Nutrition and Efficient Feed Utilization Key Laboratory of Sichuan Province, Sichuan Agricultural University, Chengdu 611130, China; Animal Nutrition Institute, Animal Nutrition and Efficient Feed Utilization Key Laboratory of Sichuan Province, Sichuan Agricultural University, Chengdu 611130, China; Animal Nutrition Institute, Animal Nutrition and Efficient Feed Utilization Key Laboratory of Sichuan Province, Sichuan Agricultural University, Chengdu 611130, China; Animal Nutrition Institute, Animal Nutrition and Efficient Feed Utilization Key Laboratory of Sichuan Province, Sichuan Agricultural University, Chengdu 611130, China; Animal Nutrition Institute, Animal Nutrition and Efficient Feed Utilization Key Laboratory of Sichuan Province, Sichuan Agricultural University, Chengdu 611130, China; Animal Nutrition Institute, Animal Nutrition and Efficient Feed Utilization Key Laboratory of Sichuan Province, Sichuan Agricultural University, Chengdu 611130, China; Animal Nutrition Institute, Animal Nutrition and Efficient Feed Utilization Key Laboratory of Sichuan Province, Sichuan Agricultural University, Chengdu 611130, China; Animal Nutrition Institute, Animal Nutrition and Efficient Feed Utilization Key Laboratory of Sichuan Province, Sichuan Agricultural University, Chengdu 611130, China; Animal Nutrition Institute, Animal Nutrition and Efficient Feed Utilization Key Laboratory of Sichuan Province, Sichuan Agricultural University, Chengdu 611130, China; Animal Nutrition Institute, Animal Nutrition and Efficient Feed Utilization Key Laboratory of Sichuan Province, Sichuan Agricultural University, Chengdu 611130, China

**Keywords:** all-trans retinoic acid, antivirus, apoptosis, diarrhea, intestinal health, piglet

## Abstract

Transmissible gastroenteritis virus (TGEV) is a significant pathogen responsible for diarrhea in piglets, with its pathogenesis intricately associated with apoptosis. As a bioactive vitamin A derivative, all-trans retinoic acid (ATRA) possesses antiviral, antioxidant and anti-apoptotic characteristics. This research aimed to explore whether ATRA could mitigate TGEV infection in piglets through inhibition of intestinal epithelial apoptosis. In a 19-day trial, 32 piglets were randomized into four treatments: non-challenged group (Control), TGEV-challenged group (TGEV), TGEV+ 5 mg/d ATRA group (TGEV+ATRA5) and TGEV+ 15 mg/d ATRA group (TGEV+ATRA15). On day 15, piglets except the control group were challenged with TGEV. Results demonstrated that piglets administered with ATRA effectively prevented growth inhibition, diarrhea and intestinal impairment caused by TGEV, as confirmed by increased average daily gain, reduced diarrhea rate and diarrhea score, and upregulated villus height and tight-junction protein level (ZO-1 and Occludin) (*P *< 0.05). ATRA also markedly decreased TGEV RNA copies in the jejunum of TGEV-infected piglets (*P *< 0.05). Meanwhile, ATRA inhibited TGEV-induced intestinal epithelial apoptosis through inhibition of apoptosis-related protein expression (Bax, Fas, Caspase-9, -8, and -3) (*P *< 0.05). In addition, ATRA markedly attenuated TGEV-induced oxidative stress through reducing the production of hydrogen peroxide (H_2_O_2_) and malondialdehyde (MDA), while improving the activities of antioxidant enzymes (GSH-PX, SOD, and CAT) (*P *< 0.05). Further research found that ATRA also down-regulated the phosphorylation levels of P_38_MAPK and JNK, as well as the levels of apoptosis pathway-related proteins (GRP78, p-PERK, ATF6, and CHOP) mediated by endoplasmic reticulum stress (ERS) in jejunal mucosa of TGEV-infected piglets (*P *< 0.05). Our research demonstrated that ATRA could mitigate TGEV-triggered diarrhea and intestinal impairment of piglets by suppressing TGEV replication and intestinal epithelial apoptosis. The underlying mechanism by which ATRA inhibits intestinal epithelial apoptosis may be related to the suppression of oxidative stress-mediated P_38_MAPK/JNK pathways and ERS-mediated PERK/ATF6-CHOP pathways.

## Graphical Abstract

**Graphical Abstract:**
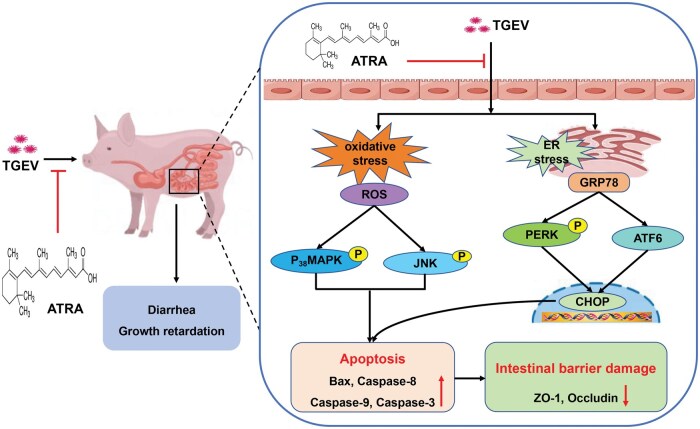

## Teaser

ATRA attenuates TGEV-induced intestinal impairment of piglets by suppressing TGEV replication and intestinal epithelial apoptosis, thereby inhibiting diarrhea and improving growth performance.

## Introduction

Transmissible gastroenteritis virus (TGEV) is a major pathogen responsible for causing diarrhea and death in piglets, with mortality rates reaching as high as 100% in those under 2 wk old (Yang et al., 2018). The intestine of pigs is the primary organ infected by TGEV. TGEV invades and proliferates within intestinal epithelial cells, resulting in intestinal villi atrophy and damage to the intestinal barrier structure. This damage reduces the intestinal digestion and absorption capacity, ultimately leading to severe diarrhea and mortality in piglets, which seriously threatens the healthy growth of the swine industry (Pu et al., 2024). Due to the genetic variability of TGEV, current conventional vaccines are unable to provide effective protection for pigs (Tuboly et al., 2000). Consequently, it is imperative to identify and develop new anti-TGEV agents.

Growing evidence indicated that the pathogenic mechanism of virus is strongly linked to apoptosis, where ROS-mediated oxidative stress and endoplasmic reticulum stress (ERS) have been identified as key factors in the induction of cell apoptosis by viruses. For example, PEDV infection triggers ROS accumulation in Vero cells, thus activating the p53 signaling pathway and inducing apoptosis (Xu et al., 2019). Rotavirus infection triggered ERS-mediated apoptosis in IPEC-J2 cells via activating the PERK-eIF2α signaling (Zhao et al., 2021). Earlier research found that TGEV infection can initiate death receptors and mitochondrial mediated pathways to trigger apoptosis, which subsequently causes cytopathic effects and the demise of host cells (Ding et al., 2012). In alignment with the pathological alterations in vitro, infection with TGEV notably raised the level of pro-apoptosis protein Caspase-3 in the jejunum of piglets, and destroyed the intestinal morphology and structure (Chai et al., 2014). Furthermore, studies indicate that ROS participates in apoptosis induced by TGEV. It was documented that TGEV stimulated the production of ROS, thus inducing apoptosis in Pk-15 cells (Ding et al., 2018). Our prior research has established that TGEV infection triggered IPEC-J2 cell apoptosis through P_38_MAPK pathways mediated by ROS, which led to cell damage (Pu et al., 2022). These findings confirm the pivotal role of apoptosis in TGEV pathogenesis, suggesting that targeting intestinal epithelial apoptosis could be an effective approach to mitigate TGEV infection.

As a bioactive vitamin A derivative, all-trans retinoic acid (ATRA) exhibits diverse functions including anti-inflammation, antiviral, enhancement of vision, and modulation of cellular differentiation (Mora et al., 2008; Gonçalves et al., 2024). Recent research has elucidated that ATRA is also pivotal in the modulation of apoptosis, potentially linked to its strong antioxidant capacity. It was reported that ATRA alleviated doxorubicin-induced cardiomyocyte apoptosis in rats by improving antioxidant capacity (Khafaga and El-Sayed, 2018). Previous research has shown that ATRA prevents apoptosis in cardiomyocytes caused by angiotensin II and mechanical stretch through the inhibition of ROS production (Choudhary et al., 2008). Chatterjee et al. discovered that ATRA mitigated arsenic‐induced apoptosis in the uterus of rat by suppressing ROS-mediated MAPK pathway (Chatterjee and Chatterji, 2017). In addition, ATRA can also play an anti-apoptosis role by inhibiting ERS. Prior research has indicated that ATRA can alleviate ethanol-induced hepatocyte apoptosis of rats through the inhibition of ERS (Nair et al., 2018). Zhou et al. demonstrated that ATRA can inhibit apoptosis in brain microvascular endothelial cells mediated by ERS and prevent the impairment of blood–spinal cord barrier subsequent to spinal cord damage (Zhou et al., 2016). Collectively, these findings indicate that ATRA possesses anti-apoptosis characteristics. Nevertheless, the role of ATRA in TGEV-induced intestinal epithelial apoptosis of piglets remains unclear. Thus, this research explored ATRA’s protective role and mechanism against TGEV-triggered intestinal epithelial apoptosis and intestinal barrier impairment in piglets. Our research demonstrated that ATRA can attenuate TGEV-triggered intestinal impairment by suppressing TGEV replication and intestinal epithelial apoptosis, thereby inhibiting diarrhea in piglets.

## Materials and Methods

This research was conducted in compliance with the protocols sanctioned by the Animal Ethical Committee of Sichuan Agricultural University (CD-SYXK-2017-015).

### Materials

TGEV strain SC-Y (GenBank accession no. DQ443743) was obtained from Sichuan Agricultural University’s Veterinary Medicine College. ATRA (R2625, purity ≥ 98%) was sourced from Sigma (USA).

### Experimental design

**Figure 1.**
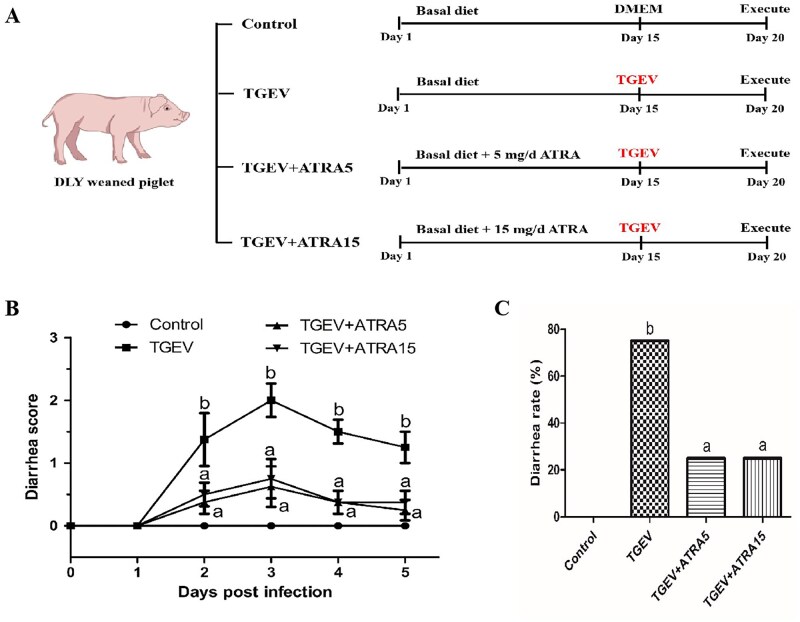
Effects of ATRA on diarrhea of TGEV-infected piglets. (A) Animal experiment design. (B and C) The diarrhea score and diarrhea rate of piglets (post-infection). ^a,b^ Within a row, different letters indicate significant difference (*P *< 0.05, *n* = 8).

Thirty-two healthy TGEV-seronegative weaned piglets at 25 d of age (16 barrows and 16 gilts) with initial body weight (BW) of 7.63 ± 0.33 kg were assigned to four treatments (*n* = 8) based on a randomized complete block design with sex and BW as blocks. The treatment groups including: Control, TGEV, TGEV+ATRA5, and TGEV+ATRA15. This research was designed as illustrated in Figure 1A. Piglets were provided with basal diet and administered with different doses of ATRA (0, 0, 5, and 15 mg/d) for 19 days. The basal diet (Supplementary Table S1) for piglets was formulated to meet nutrient requirements of weaned piglets. On day 15 of the experiment, piglets in all groups except the control were orally administered with 200 ml of TGEV (1 × 10^6.85^ TCID_50_/mL), while the control group was administered an equivalent amount of sterile medium. Following the TGEV challenge, the fecal consistency of piglets was scored daily to evaluate diarrhea severity. Fecal consistency was categorized into four levels: 0, normal; 1, pasty; 2, semiliquid; 3, liquid (Pu et al., 2020). The mean daily diarrhea score was calculated by dividing the sum of scores per group by the number of piglets in the group. Piglets were ­considered as diarrhea when the fecal consistency score is ≥2. Diarrhea rate (%) = (Number of piglets with diarrhea/Total number of piglets) ×100. Body weights of piglets on days 1 and 20, along with daily feed consumption were documented. These data were utilized to analyze growth performance indicators such as ADG, ADFI, and G:F. During the entire experiment, piglets were individually maintained in metabolic cages within a temperature-regulated nursery (25–28°C), with free access to water and feed.

### Sample collection

Blood was obtained from each piglet’s anterior vena cava on day 20. After centrifugation (3500×*g*, 15 min), the serum was isolated and frozen at −20°C for later analysis. Subsequently, the piglets were euthanized to collect intestinal samples. The middle segments of the jejunum were obtained and subsequently fixed in 4% paraformaldehyde for TUNEL assay and histological analysis. Meanwhile, the jejunal mucosa was scraped and frozen at −80°C until analysis.

### Histological analysis

Jejunal tissue samples were fixed in 4% paraformaldehyde solution, then dehydrated, embedded, and sliced. Subsequently, the sections were processed for hematoxylin–eosin (HE) staining and examined under a light microscope (Eclipse Ci-L, China). Crypt depth and villus height were quantified with Image-Pro Plus 6.0 software.

### Analysis of antioxidant parameters

Antioxidant parameters in serum and jejunal mucosa of piglets, including catalase (CAT), glutathione peroxidase (GSH-PX), superoxide dismutase (SOD), total antioxidant capacity (T-AOC), malondialdehyde (MDA), hydrogen peroxide (H_2_O_2_), and nitric oxide (NO) were analyzed by corresponding assay kits following the guidelines of the manufacturer (Jiancheng Bioengineering Institute, Nanjing, China).

### Detection of apoptosis

The apoptosis in jejunum samples was assessed by TUNEL test as outlined by Li et al. (2018). Briefly, paraffin-embedded jejunum sections underwent dewaxed, dehydrated, and permeabilized using proteinase K. Next, apoptosis was detected using Servicebio’s TUNEL kit (Wuhan, China). Ultimately, TUNEL-positive cells were identified by fluorescence microscope (Nikon, Tokyo, Japan). Apoptotic index (%) = 100 × (TUNEL-positive cells/total cells).

### Western blot analysis

Firstly, based on the two key clinical indicators of diarrhea score and average daily weight gain for each treatment group, representative individuals whose phenotypes were closest to the average of the group were screened out; subsequently, from the individuals that met the above criteria in each treatment group, three jejunal mucosa samples from piglets were randomly selected for western blot analysis. The jejunal mucosa was lysed in RIPA buffer (Beyotime, China), and the protein concentration was quantified by BCA kit (Thermo, USA). The same amount of protein was then subjected to SDS-PAGE for separation and transferred onto PVDF membrane. Following 1 h blocking with 5% non-fat dry milk, membranes were sequentially incubated with primary and corresponding secondary antibodies (Supplementary Table S2). Subsequently, the blots were detected using a ChemiDoc^TM^ Imager (Bio-Rad) and analyzed with Image Lab software.

### qRT-PCR analysis

Jejunal mucosal RNA was extracted using Trizol reagent (TaKaRa, China), with 1 µg RNA reverse transcribed into cDNA (PrimeScript RT kit, TaKaRa, China). The relative quantification qRT-PCR was conducted on the QuantStudio 5 Flex system (Bio-Rad, USA) with SYBR kits. The primer sequence was listed in Supplementary Table S3. Data were normalized to β-actin and calculated through the 2^−ΔΔCt^ method (Livak and Schmittgen, 2001). Absolute quantification qRT-PCR was employed to quantify the copies of TGEV RNA in the jejunal mucosa. The sequences of probes and primers for TGEV-N gene are listed in Supplementary Table S4. The recombinant plasmid containing the TGEV-N gene (Sangon Biotech, Shanghai, China) was serially diluted and utilized to generate a standard curve for quantitative analysis. The copies of TGEV RNA were calculated based on the standard curve.

### Statistical analysis

Experimental data, except diarrhea rate, were evaluated using one-way ANOVA in SPSS 22.0 (SPSS Inc., USA) with Tukey’s test for multiple comparisons. The diarrhea rate was analyzed with the Chi-square test. Results are presented as means ± standard errors (SE). Differences were deemed significant when *P *< 0.05.

## Results

### Growth performance and diarrhea

Compared to control group, TGEV-infected piglets showed an 8.88% decrease in ADG. However, treatment with 5 mg/d ATRA markedly reversed TGEV-induced growth inhibition (Table 1, *P *< 0.05). Furthermore, TGEV infection substantially induced severe diarrhea in piglets, whereas treatment with 5 and 15 mg/d ATRA markedly decreased both diarrhea severity (Figure 1B, *P *< 0.05) and diarrhea rate (Figure 1C, *P *< 0.05).

**Table 1.** Effects of ATRA on growth performance of TGEV-infected piglets

Items	Treatments				*P-*value
	Control	TGEV	TGEV+ATRA5	TGEV+ATRA15	
**ADFI, g/d**	463 ± 31.9	436 ± 26.8	516 ± 26.0	465 ± 16.0	0.202
**ADG, g/d**	304 ± 17.1^a,b^	277 ± 18.6^a^	348 ± 16.6^b^	300 ± 12.5^a,b^	0.034
**G: F**	0.66 ± 0.02	0.63 ± 0.02	0.68 ± 0.01	0.65 ± 0.02	0.345

a, b Within a row, different letters indicate significant difference (*P *< 0.05, *n* = 8).

### TGEV RNA copies in jejunal mucosa

**Figure 2.**
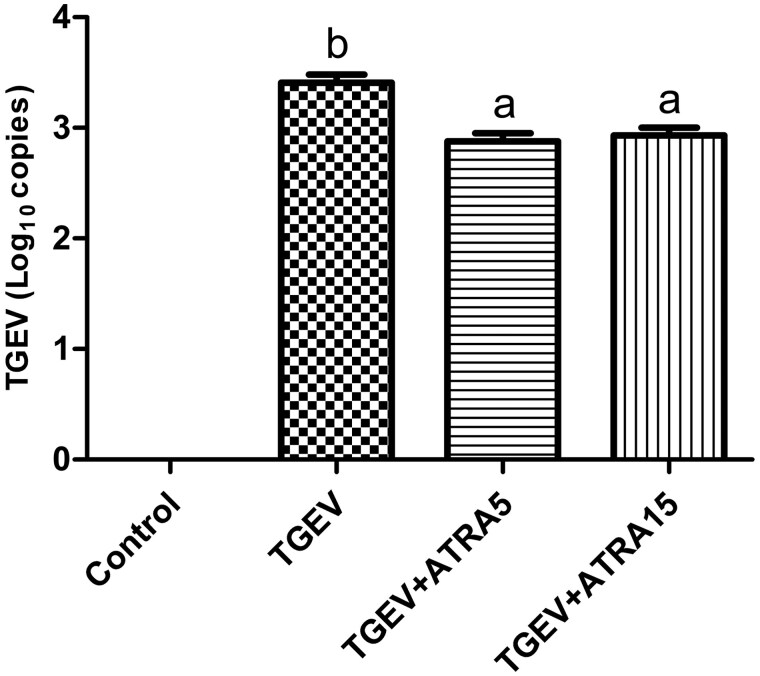
Effects of ATRA on TGEV RNA copies in jejunal mucosa of TGEV-infected piglets. ^a, b^ Within a row, different letters indicate significant difference (*P *< 0.05, *n* = 8).

As shown in Figure 2, TGEV genes were highly expressed in jejunal mucosa of all TGEV-infected piglets, whereas no TGEV genes were detected in the control group. Furthermore, TGEV RNA copies in jejunal mucosa of TGEV+ATRA5 and TGEV+ATRA15 groups were significantly lower than that in the TGEV group (*P *< 0.05).

### Intestinal morphology

**Figure 3.**
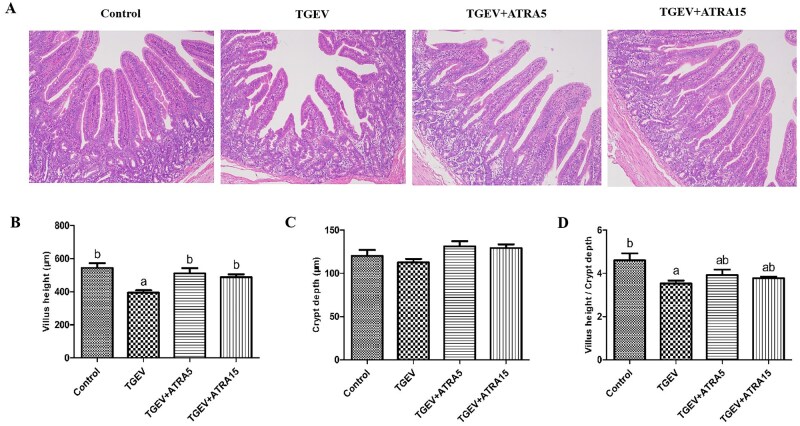
Effects of ATRA on intestinal morphology of TGEV-infected piglets. (A) Representative HE-stained jejunum sections (×100). (B–D) Villus height, crypt depth and villus height-to-crypt depth ratio was quantified. ^a, b^ Within a row, different letters indicate significant difference (*P *< 0.05, *n* = 8).

As shown in Figure 3, TGEV infection markedly reduced villus height and the villus height-to-crypt depth ratio in the jejunum of piglets compared with the control group (*P *< 0.05). However, treatment with 5 and 15 mg/d ATRA markedly attenuated TGEV-triggered reductions in jejunal villus height (*P *< 0.05).

### Intestinal barrier integrity

**Figure 4.**
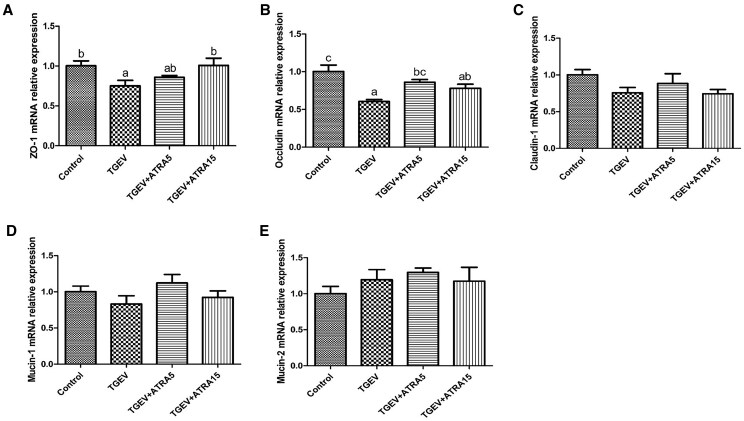
Effects of ATRA on intestinal barrier integrity of TGEV-infected piglets. (A–E) The mRNA expressions of ZO-1, Occludin, Claudin-1, Mucin-1, and Mucin-2 in jejunal mucosa. ^a, b, c^ Within a row, different letters indicate significant difference (*P *< 0.05, *n* = 8).

As illustrated in Figure 4, TGEV infection caused a notable decrease in ZO-1 and Occludin mRNA expression in jejunal mucosa relative to control (*P *< 0.05). While piglets administered with 5 mg/d ATRA markedly attenuated TGEV-triggered downregulation of Occludin mRNA expression (*P *< 0.05). Meanwhile, treatment with 15 mg/d ATRA markedly suppressed TGEV-triggered decreases in ZO-1 mRNA expression (*P *< 0.05).

### Intestinal epithelial apoptosis

**Figure 5.**
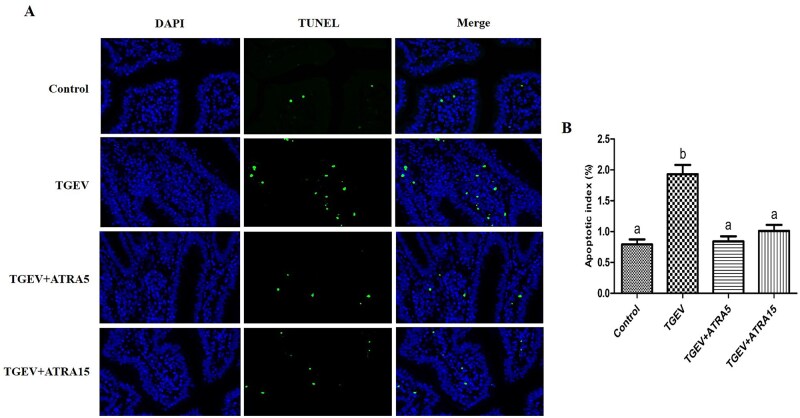
Effects of ATRA on intestinal epithelial apoptosis of TGEV-infected piglets. (A) Representative TUNEL stained images for detection of jejunal epithelial cells apoptosis (×400). (B) Quantification of TUNEL-stained cell numbers. ^a, b^ Within a row, different letters indicate significant difference (*P *< 0.05, *n* = 8).

**Figure 6.**
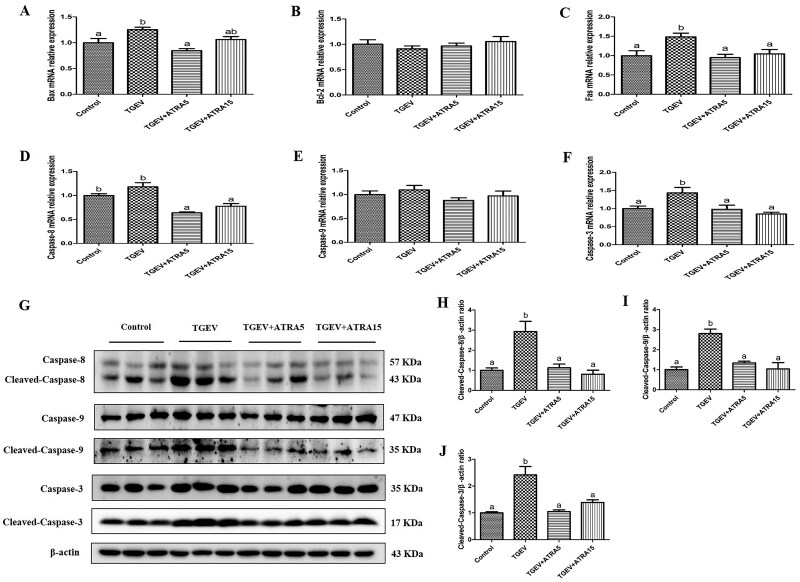
Effects of ATRA on the apoptosis-related genes and proteins expressions in jejunal mucosa of TGEV-infected piglets. (A–F) The mRNA expressions of apoptosis-related genes (*n* = 8). (G–J) The levels of apoptosis-related proteins were analyzed by western blot (*n* = 3). ^a, b^ Within a row, different letters indicate significant difference (*P *< 0.05).

TUNEL analysis demonstrated that infection with TGEV markedly increased the apoptotic index of jejunal epithelial cells, while supplementation with 5 and 15 mg/d ATRA markedly suppressed TGEV-induced jejunal epithelial apoptosis (Figure 5, *P *< 0.05). To further validate the anti-apoptosis role of ATRA, we assessed the jejunal mucosa for changes in apoptosis-associated gene and protein expression. As illustrated in Figure 6, administration of 5 and 15 mg/d ATRA markedly attenuated the upregulation of Bax, Fas, Caspase-8, and Caspase-3 mRNA expression, as well as the elevation of cleaved-caspase-8, cleaved-caspase-9, and cleaved-caspase-3 protein levels triggered by TGEV (*P *< 0.05).

### Intestinal oxidative stress

Oxidative stress is an unbalanced state between antioxidant defense and ROS generation (Forman and Zhang, 2021). To evaluate ATRA’s role in mitigating TGEV-triggered intestinal oxidative stress, we measured the levels of ROS (NO and H_2_O_2_) and MDA and antioxidant enzyme activities (Table 2). In comparison to the control treatment, TGEV infection remarkably elevated the concentration of H_2_O_2_ in jejunal mucosa, whereas administration of 5 and 15 mg/d ATRA notably suppressed TGEV-induced the increase in H_2_O_2_ concentration (*P *< 0.05). Furthermore, compared to controls, TGEV infection notably reduced SOD and GSH-PX activities in serum, along with SOD, GSH-PX, and CAT activities in jejunal mucosa, while elevating MDA contents in both serum and jejunal mucosa (*P *< 0.05). Nevertheless, administration of 5 mg/d ATRA effectively mitigated the reduction of SOD activity in serum and SOD and GSH-PX activities in jejunal mucosa, and the increase of MDA content in jejunal mucosa caused by TGEV (*P *< 0.05). Meanwhile, the administration of 15 mg/d ATRA markedly mitigated TGEV-induced the reduction of GSH-PX and CAT activities in jejunal mucosa (*P *< 0.05).

**Table 2.** Effects of ATRA on antioxidant capacity of TGEV-infected piglets

Items	Treatments				*P*-value
	Control	TGEV	TGEV+ATRA5	TGEV+ATRA15	
*Serum*					
**GSH-PX, U/mL**	530.73 ± 11.71^b^	466.79 ± 14.87^a^	497.99 ± 16.80^a,b^	479.93 ± 15.82^a,b^	0.030
**SOD, U/mL**	71.05 ± 1.85^b^	56.34 ± 2.63^a^	67.40 ± 3.18^b^	62.11 ± 1.36^a,b^	0.001
**CAT, U/mL**	8.42 ± 0.46	7.86 ± 1.03	8.60 ± 0.51	8.77 ± 0.49	0.918
**T-AOC, mmol/mL**	0.51 ± 0.02	0.49 ± 0.01	0.52 ± 0.01	0.51 ± 0.01	0.102
**MDA, nmol/mL**	2.86 ± 0.18^a^	3.68 ± 0.25^b^	3.17 ± 0.12^a,b^	3.25 ± 0.12^a,b^	0.025
***Jejunal mucosa***					
**GSH-PX, U/mg prot**	36.00 ± 4.76^b^	23.75 ± 2.17^a^	35.68 ± 1.11^b^	38.55 ± 2.26^b^	0.006
**SOD, U/mg prot**	3.60 ± 0.28^b^	2.51 ± 0.23^a^	3.48 ± 0.18^b^	3.26 ± 0.18^a,b^	0.008
**CAT, U/mg prot**	12.82 ± 1.30^b^	7.65 ± 0.48^a^	10.19 ± 0.45^a,b^	10.90 ± 0.62^b^	0.001
**T-AOC, mmol/mg prot**	0.12 ± 0.01	0.10 ± 0.01	0.11 ± 0.01	0.10 ± 0.01	0.350
**MDA, nmol/mg prot**	1.25 ± 0.15^a^	1.93 ± 0.16^b^	1.40 ± 0.07^a^	1.57 ± 0.06^a,b^	0.003
**NO, umol/g prot**	0.10 ± 0.02	0.18 ± 0.04	0.16 ± 0.01	0.15 ± 0.02	0.157
**H_2_O_2_, mmol/g prot**	5.69 ± 0.31^a^	8.98 ± 0.41^b^	5.96 ± 0.25^a^	6.20 ± 0.33^a^	0.001

a, b Within a row, different letters indicate significant difference (*P *< 0.05, *n* = 8).

### MAPK signaling pathway in jejunal mucosa

**Figure 7.**
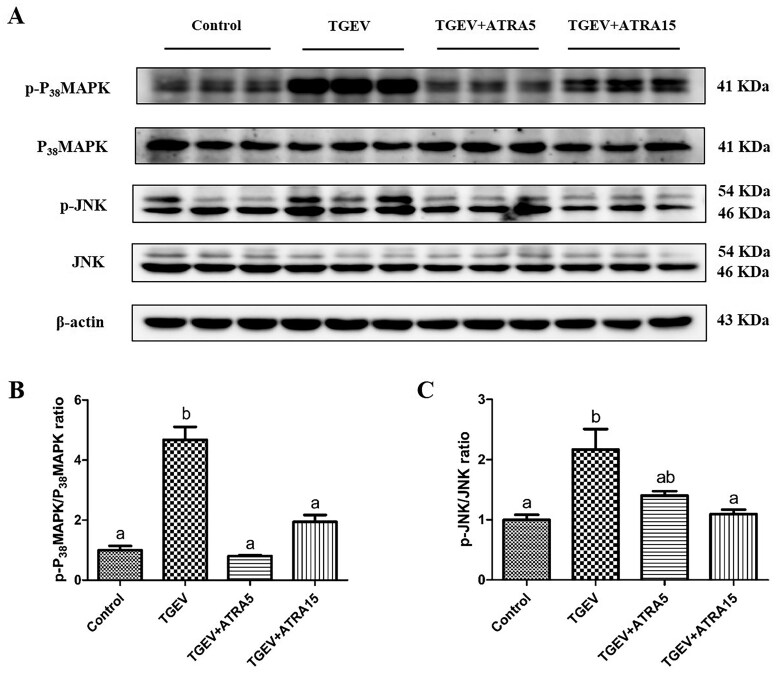
Effects of ATRA on MAPK pathway in jejunal mucosa of TGEV-infected piglets. (A–C) The protein levels of p-P_38_MAPK and p-JNK were analyzed by western blot. ^a, b^ Within a row, different letters indicate significant difference (*P *< 0.05, *n* = 3).

As illustrated in Figure 7, the phosphorylation levels of P_38_MAPK and JNK in jejunal mucosa of TGEV-infected piglets were markedly higher than that in the control treatment (*P *< 0.05). Nevertheless, administration of 5 mg/d ATRA remarkably mitigated the increase in P_38_MAPK phosphorylation level caused by TGEV (*P *< 0.05). Concurrently, administration of 15 mg/d ATRA notably blocked TGEV-induced the elevation of P_38_MAPK and JNK phosphorylation level (*P *< 0.05).

### ERS-mediated apoptosis pathways in jejunal mucosa

**Figure 8.**
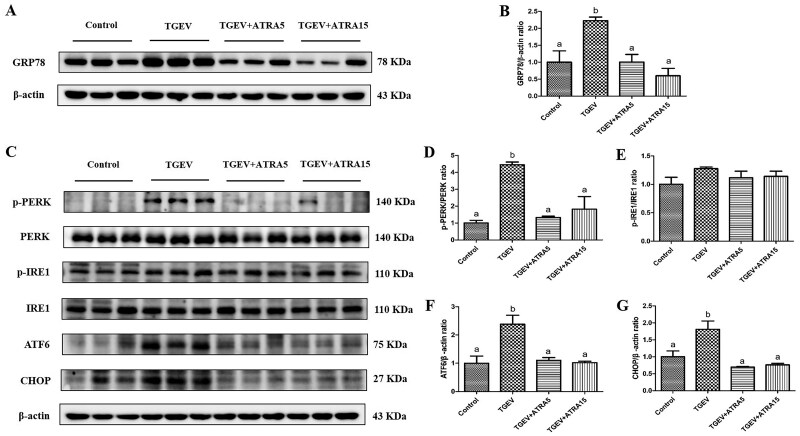
Effects of ATRA on ERS-mediated apoptosis pathways in jejunal mucosa of TGEV-infected piglets. (A–G) The protein levels of GRP78, p-PERK, p-IRE1, ATF6, and CHOP were analyzed by western blot. ^a, b^ Within a row, different letters indicate significant difference (*P *< 0.05, *n* = 3).

To investigate whether ATRA can alleviate intestinal epithelial apoptosis caused by TGEV through the inhibition of ERS, we measured the levels of ERS-mediated apoptosis pathways related proteins. As illustrated in Figure 8, infection with TGEV remarkably elevated the levels of GRP78, ATF6, CHOP protein, and PERK phosphorylation level in jejunal mucosa (*P *< 0.05). Nevertheless, administration of 5 and 15 mg/d ATRA remarkably mitigated TGEV-induced the elevation of GRP78, ATF6, and CHOP protein levels and PERK phosphorylation level (*P *< 0.05).

## Discussion

The intestinal barrier integrity is crucial for the health of both animals and humans (Wang et al., 2023). TGEV is a porcine enteropathogenic coronavirus, which can cause intestinal barrier damage and dysfunction, leading to severe diarrhea and elevated mortality rates in piglets (Pu et al., 2024). Apoptosis, often termed programmed cell death, plays a key role in preserving normal tissue homeostasis, but excessive apoptosis will lead to intestinal barrier dysfunction (Zhang et al., 2022). Previous research has demonstrated that TGEV (Shaanxi strain) infection can trigger apoptosis by activating pro-apoptosis signals, and further lead to host cell damage and death in vitro (Huang et al., 2013). In alignment with the pathological alterations in vitro, TGEV (Purdue 46-MAD strain and TS strain) infection also triggered jejunal epithelial apoptosis in piglets, which damaged the intestinal barrier structure and function, ultimately leading to severe diarrhea and growth inhibition of piglets (Chai et al., 2014; Wang et al., 2022). In line with these findings, our research also confirmed that infection with TGEV (SC-Y strain) promoted apoptosis of jejunal epithelial cells, provoked intestinal villus atrophy and intestinal barrier damage, and caused diarrhea in piglets. These studies indicate that different TGEV strains share a high degree of similarity in their pathogenic mechanisms, all capable of inducing cell apoptosis and villus atrophy, ultimately leading to intestinal barrier damage and severe diarrhea. Therefore, suppressing the apoptosis of intestinal epithelial cells may represent a viable strategy to alleviate TGEV infection in piglets.

ATRA is a bioactive natural derivative of vitamin A, exhibiting diverse functions including anti-inflammation, enhancement of vision, antiviral, and modulation of cellular differentiation (Mora et al., 2008; Gonçalves et al., 2024). Recent research has elucidated that ATRA possesses both antioxidant and antiapoptotic characteristics. Zhang et al. demonstrated that ATRA exerts anti-apoptotic effects on oxygen-glucose deprived PC12 cells through the RARα signaling pathway (Zhang et al., 2014). Previous research demonstrated that ATRA effectively mitigated cardiomyocyte apoptosis caused by angiotensin II and mechanical stretch through the inhibition of ROS generation and the enhancement of the antioxidant capacity (Choudhary et al., 2008). Nevertheless, it remains uncertain whether ATRA can mitigate intestinal epithelial apoptosis in piglets caused by TGEV. In the current investigation, we observed that piglets administered with 5 and 15 mg/d of ATRA evidently mitigated intestinal epithelial apoptosis, intestinal villus atrophy and intestinal barrier impairment caused by TGEV, as demonstrated by decreased the apoptotic index of jejunal epithelial cells and elevated villus height and the abundance of tight junction proteins (ZO-1 and Occludin) in the jejunum. Furthermore, feeding with ATRA also markedly mitigated diarrhea and growth inhibition of piglets caused by TGEV and decreased TGEV RNA copies in the jejunum of TGEV-infected piglets. Among them, the 5 mg/d ATRA group was superior to the 15 mg/d ATRA group in improving the ADG of piglets, which might be related to the inhibitory effect of high-dose ATRA on feeding behavior. The data of this study showed that the ADFI of piglets in the 15 mg/d ATRA group was 9.9% lower than that in the 5 mg/d group. These findings suggest that ATRA can mitigate TGEV-induced intestinal barrier impairment through suppressing TGEV replication and intestinal epithelial apoptosis, thereby reducing diarrhea and enhancing the growth performance of piglets.

Apoptosis is mainly initiated through two pathways, including the death receptor pathway and the mitochondrial pathway. The death receptor pathway is typically initiated by the ligand from the TNF family death receptors, which subsequently activates caspase-8 (Takata et al., 2015). The mitochondrial pathway is modulated by Bcl-2 family members, which modulate mitochondrial membrane permeability, thus causing caspase-9 activation (You et al., 2019). Both of these pathways activate caspase-3, a key effector molecule in apoptosis, thus triggering apoptosis. Prior research has demonstrated that infection with TGEV can trigger PK-15 cell apoptosis via the activation of both mitochondrial and death receptor apoptotic pathways (Ding et al., 2012). To validate ATRA’s protective role against TGEV-triggered intestinal epithelial apoptosis, we assessed the expression of apoptosis-related markers. Our findings indicate that ATRA administration markedly suppressed the upregulation of mRNA expressions for Bax, Fas, Caspase-3, and Caspase-8, as well as the elevation in protein levels of Cleaved-caspase-8, Cleaved-caspase-9, and Cleaved-caspase-3 in the jejunal mucosa of piglets caused by TGEV. These findings suggest that ATRA could mitigate TGEV-triggered intestinal epithelial apoptosis through the inhibition of mitochondrial and death receptor-mediated apoptotic pathways.

Numerous studies have established that ROS-induced oxidative stress serves as a pivotal regulator in virus-induced apoptosis (Forman and Zhang, 2021). For example, infection with porcine parvovirus resulted in the generation of ROS in ST cells, subsequently triggering the mitochondrial apoptosis pathway and inducing apoptosis (Zhao et al., 2016). Similar to prior studies, our research found that piglets infected with TGEV markedly elevated the concentration of ROS (such as H_2_O_2_) in jejunal mucosa. Nevertheless, ATRA administration markedly attenuated the elevation of ROS concentration caused by TGEV, which may be related to ATRA’s strong antioxidant capacity. Khafaga et al. demonstrated that ATRA mitigated cardiomyocyte apoptosis caused by doxorubicin in rats through the enhancement of antioxidant enzyme activities (SOD, GSH-PX, and CAT) (Khafaga and El-Sayed, 2018). Our research found that administration of 5 mg/d ATRA markedly mitigated the reduction in serum SOD activity, and SOD and GSH-PX activities in jejunal mucosa, and the elevation of MDA content within the jejunal mucosa caused by TGEV, which was aligned with the findings that ATRA can mitigate ROS production caused by TGEV and prevent intestinal epithelial apoptosis. The aforementioned findings suggest that ATRA could mitigate TGEV-induced intestinal epithelial apoptosis through the inhibition of oxidative stress. In addition, a multitude of studies has demonstrated that the MAPK pathway is pivotal in mediating apoptosis caused by oxidative stress (Zhang et al., 2020). For instance, infection with PRRSV induced Marc-145 cells apoptosis through the ROS-dependent JNK pathway (Shao et al., 2022). Ding et al. demonstrated that infection of TGEV triggers PK-15 cells apoptosis via the activation of ROS-dependent P_38_MAPK and p53 pathways (Ding et al., 2013). Our research found that infection with TGEV markedly upregulated the phosphorylation levels of P_38_MAPK and JNK in jejunal mucosa of piglets. Nevertheless, ATRA administration markedly inhibited TGEV-induced increase in P_38_MAPK and JNK phosphorylation levels. These findings demonstrated that ATRA may mitigate TGEV-induced intestinal epithelial apoptosis through suppressing oxidative stress-mediated P_38_MAPK/JNK signaling pathways.

Besides oxidative stress, ERS also plays a crucial role in apoptosis triggered by viral infections (Wang et al., 2019). ERS is a cell state in which protein folding ability is destroyed. When ER homeostasis is disrupted, excessive unfolded proteins triggers glucose-regulated protein 78 (GRP78) release from ER transmembrane proteins, and activate the unfolded protein response (UPR) to remove misfolded or unfolded proteins, thus alleviating ERS (Wang and Li, 2019). The UPR is driven by three transmembrane proteins, namely IRE1, PERK, and ATF6 (Prasad and Greber, 2021). However, whether ERS is too severe to recover, the overloaded UPR will activate the apoptosis process through its downstream transcription factor (CHOP) (Hetz et al., 2020). Earlier research has demonstrated that porcine parvovirus infection induced ERS-mediated apoptosis in PK-15 cells through activating the PERK–CHOP pathway (Cao et al., 2020). Chen et al. demonstrated that co-infection with PRV and PCV2 can initiate ERS through the IRE1-XBP1-EDEM and PERK-eIF2α-CHOP pathways, subsequently leading to apoptosis in PK-15 cells (Chen et al., 2022). Xue et al. demonstrated that infection with TGEV can trigger ERS in IPEC-J2 cells, which is manifested by the increase of GRP78 protein level (Xue et al., 2018). Our present investigation revealed that infection with TGEV remarkably increased the protein levels of GRP78, ATF6, CHOP, as well as PERK phosphorylation level in jejunal mucosa. These findings suggest that TGEV could trigger apoptosis of intestinal epithelial cells mediated by ERS via the activation of the PERK/ATF6–CHOP pathways. Furthermore, prior research has demonstrated that ATRA can mitigate ethanol-induced hepatocyte apoptosis in rats through suppressing ERS (Nair et al., 2018). Our study found that piglets administered with 5 and 15 mg/d ATRA markedly mitigated the elevation of GRP78, ATF6, and CHOP protein levels and PERK phosphorylation caused by TGEV. The aforementioned findings demonstrated that ATRA could mitigate TGEV-triggered intestinal epithelial apoptosis in piglets through suppressing the ERS-mediated PERK/ATF6–CHOP pathways.

In summary, our research demonstrated that ATRA can attenuate TGEV-triggered diarrhea and intestinal impairment in piglets by suppressing TGEV replication and intestinal epithelial apoptosis. The underlying mechanism by which ATRA inhibits intestinal epithelial apoptosis may be related to the suppression of oxidative stress-mediated P_38_MAPK/JNK pathways and ERS-mediated PERK/ATF6–CHOP pathways. The current research provides an effective approach for preventing TGEV infection. The TGEV SC-Y strain used in this study is a Chinese isolate. Whether ATRA has a direct antiviral effect on other TGEV strains remains unclear and requires further investigation. Although there are certain differences among different TGEV strains, they share high similarity in pathogenic mechanisms, all capable of inducing apoptosis and intestinal villus atrophy, ultimately leading to intestinal barrier damage and severe diarrhea. This study shows that the protective effect of ATRA mainly targets the common pathophysiological processes of the host rather than the characteristics of specific virus strains. This host-oriented therapeutic strategy suggests that the protective effect of ATRA may have the potential to cross strains and even pathogens—as long as the infection causes similar intestinal oxidative damage and apoptosis and other pathological changes, ATRA is expected to exert similar protective effects.

## Supplementary Material

skaf356_Supplementary_Data

## Abbreviations

ADFI: average daily feed intake
ADG: average daily gain
ATF6: activating transcription factor 6
ATRA: all-trans retinoic acid
Bax: B-cell lymphoma-2-associated X protein
Bcl-2: B-cell lymphoma-2
Caspase: cysteinyl aspartate specific proteinase
CAT: catalase
CHOP: C/EBP homologous protein
ERS: endoplasmic reticulum stress
G:F: gain-to-feed ratio
GRP78: glucose-regulated protein 78
GSH-PX: glutathione peroxidase
H_2_O_2_: hydrogen peroxide
IRE1: inositol-requiring enzyme
JNK: c-Jun N-terminal kinase
MDA: malondialdehyde
NO: nitric oxide
PERK: protein kinase-like ER kinase
P_38_MAPK: P_38_ mitogen-activated protein kinase
SOD: superoxide dismutase
T-AOC: total antioxidant capacity
TGEV: transmissible gastroenteritis virus
ZO-1: zonula occludens-1
