# Supplementary material for: All-trans retinoic acid protects piglets from TGEV-induced diarrhea and intestinal epithelial apoptosis by modulating redox status and endoplasmic reticulum stress pathways
Source: J Anim Sci. 2025 Oct 25;103:skaf356. doi: 10.1093/jas/skaf356 (PMC12602150; doi:10.1093/jas/skaf356)
Supplement: skaf356_Supplementary_Data [file skaf356_supplementary_data.zip › 2025-9-23-Supplementary material.docx]

**Supplementary Table 1**. Ingredient composition of the basal diet (%, air-dry basis)

| Ingredients | Content | Nutrient levels^3^ | Content |
| --- | --- | --- | --- |
| Corn | 30.80 | Digestible energy, MJ/kg | 14.73 |
| Extruded maize | 30.00 | Crude protein | 18.26 |
| Soybean meal | 9.00 | Calcium | 0.76 |
| Extruded soybean | 8.50 | Total phosphorus | 0.57 |
| Soybean protein concentrate | 5.20 | Available phosphorus | 0.39 |
| Sucrose | 2.00 | SID-Lysine | 1.39 |
| Soybean oil | 1.70 | SID-Methionine | 0.45 |
| Fish meal | 4.00 | SID-Threonine | 0.78 |
| Whey powder | 6.00 | SID-Tryptophan | 0.22 |
| L-Lysine-HCl | 0.45 |  |  |
| DL-Methionine | 0.14 |  |  |
| L-Threonine | 0.04 |  |  |
| L-Tryptophan | 0.02 |  |  |
| CaCO_3_ | 0.75 |  |  |
| CaHPO_4_ | 0.50 |  |  |
| Choline chloride | 0.15 |  |  |
| NaCl | 0.20 |  |  |
| Benzoic acid | 0.30 |  |  |
| Vitamin premix^1^ | 0.05 |  |  |
| Mineral premix^2^ | 0.20 |  |  |
| Total | 100.00 |  |  |

SID = standardized ileal digestibility.

^1^Vitamin premix supplied following per kilogram of diets：Vitamin E 60 IU, Vitamin D_3_ 3000 IU, Vitamin K_3_ 4.0 mg, Vitamin B_1_ 4.0 mg, Vitamin B_2_ 8.0 mg, Vitamin B_6_ 6.0 mg, Vitamin B_12_ 0.06 mg, Niacin 50 mg, Biotin 0.3 mg, Folic acid 2.0 mg, Pantothenic 30 mg.

^2^Mineral premix supplied following per kilogram of diets：Cu (CuSO_4_·5H_2_O) 120 mg, Fe (FeSO_4_·H_2_O) 100 mg, Zn (ZnSO_4_·H_2_O) 100 mg, Se (Na_2_SeO_3_) 0.3 mg, Mn (MnSO_4_·H_2_O) 20 mg, I (KI) 0.3 mg.

^3^Nutrient levels were calculated values.

**Supplementary Table 2.** Antibodies used for western blot analysis

| Antibodies | Source |
| --- | --- |
| Caspase-3 | Cell Signaling Technology (Beverly, USA) |
| Caspase-8 | Cell Signaling Technology (Beverly, USA) |
| Caspase-9 | Cell Signaling Technology (Beverly, USA) |
| P_38_MAPK | ZEN Bioscience (Chengdu, China) |
| JNK | ZEN Bioscience (Chengdu, China) |
| p-P_38_MAPK | ZEN Bioscience (Chengdu, China) |
| p-JNK | ZEN Bioscience (Chengdu, China) |
| CHOP | Cell Signaling Technology (Beverly, USA) |
| GRP78 | Abcam (Cambridge, MA, USA) |
| ATF6 | Abcam (Cambridge, MA, USA) |
| PERK | Cell Signaling Technology (Beverly, USA) |
| IRE1 | Abcam (Cambridge, MA, USA) |
| p-PERK | Cell Signaling Technology (Beverly, USA) |
| p-IRE1 | Abcam (Cambridge, MA, USA) |
| β-actin | Santa Cruz Biotechnology (Santa Cruz, USA) |

Caspase = Cysteinyl aspartate specific proteinase; JNK=c-Jun N-terminal kinase; P_38_MAPK = P_38_ mitogen-activated protein kinase; CHOP = C/EBP homologous protein; GRP78 = Glucose-regulated protein 78; ATF6 = Activating transcription factor 6; PERK = Protein kinase-like ER kinase; IRE1 = Inositol-requiring enzyme.

**Supplementary Table 3.** Primer sequences used for real-time quantitative PCR

| Gene | Primer sequence (5’–3’) | Product length (bp) | GeneBank accession |
| --- | --- | --- | --- |
| *ZO-1* | F: CGTGTCAACGCCACTATCA | 105 | XM_021098896.1 |
|  | R: TTGTCTTCCAAAGCCCCT |  |  |
| *Occludin* | F: AACTTCCACTGATGTCCCCCGT | 116 | NM_001163647.2 |
|  | R: CCTAGACTTTCCTGCTCTGCCC |  |  |
| *Claudin-1* | F: TCTTAGTTGCCACAGCATGG | 106 | NM_001244539.1 |
|  | R: CCAGTGAAGAGAGCCTGACC |  |  |
| *Mucin-1* | F: GTGCCGCTGCCCACAACCTG  R: AGCCGGGTACCCCAGACCCA | 141 | XM_021089730.1 |
| *Mucin-2* | F: GGTCATGCTGGAGCTGGACAGT  R: TGCCTCCTCGGGGTCGTCAC | 181 | XM_021082584.1 |
| *Fas* | F: TGATGCCCAAGTGACTGACC  R: GCAGAATTGACCCTCACGAT | 103 | NM_213839 |
| *Bax* | F: GACGCTGGACTTCCTTCGAG  R: GTGGCCCGAGAGAGGTTTATT | 334 | XM_013998624.2 |
| *Bcl-2* | F: GCTACTTACTGCCAAAGGGA  R: TTCAGGCGGAGCTGTAAGAG | 161 | XM_021099593.1 |
| *Caspase-3* | F: GGAATGGCATGTCGATCTGGT  R: ACTGTCCGTCTCAATCCCAC | 351 | NM_214131.1 |
| *Caspase-8* | F: TCTGCGGACTGGATGTGATT  R: TCTGAGGTTGCTGGTCACAC | 165 | XM_021074714.1 |
| *Caspase-9* | F: AATGCCGATTTGGCTTACGT  R: CATTTGCTTGGCAGTCAGGTT | 195 | XM_013998997.2 |
| *β*-actin | F: GGATGACGATATTGCTGCGC | 190 | XM_003124280.5 |
|  | R: GATGCCTCTCTTGCTCTGGG |  |  |

**Supplementary Table 4.** Primer sequences and probes used for used for real-time quantitative PCR

| Gene | Primer and probe sequence (5’ –3’) | Anneal temperature (℃) | Product length(bp) |
| --- | --- | --- | --- |
| TGEV-N | F: CCCACAACTGGCTGAATGTG  R: GGTCGCCATCTTCCTTTGAA  P: TCCATCTGTGTCTAGCATT | 60.0 | 156 |
