# Supplementary material for: All-trans retinoic acid protects piglets from TGEV-induced diarrhea and intestinal epithelial apoptosis by modulating redox status and endoplasmic reticulum stress pathways
Source: J Anim Sci. 2025 Oct 25;103:skaf356. doi: 10.1093/jas/skaf356 (PMC12602150; doi:10.1093/jas/skaf356)
Supplement: skaf356_Supplementary_Data [file skaf356_supplementary_data.zip › Original WB Image.docx]

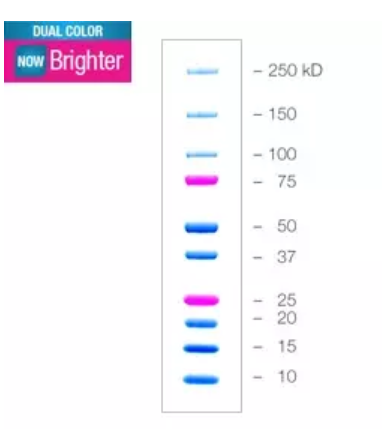
Molecular weight marker (Bio-Rad, 1610374)

**Figure 6G**


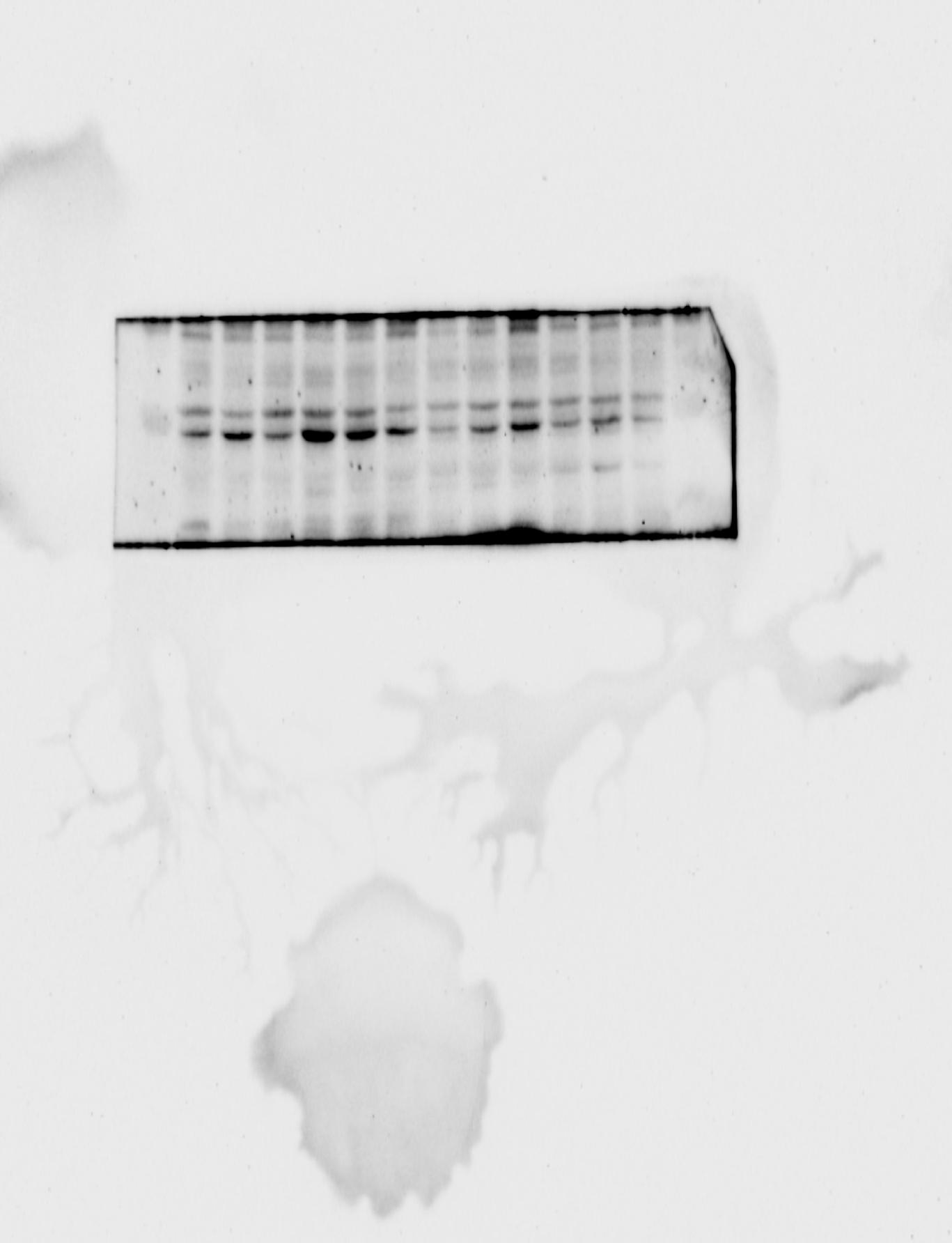


43 kDa

57 kDa

Cleaved-Caspase-8

Caspase-8

**Figure 6G**


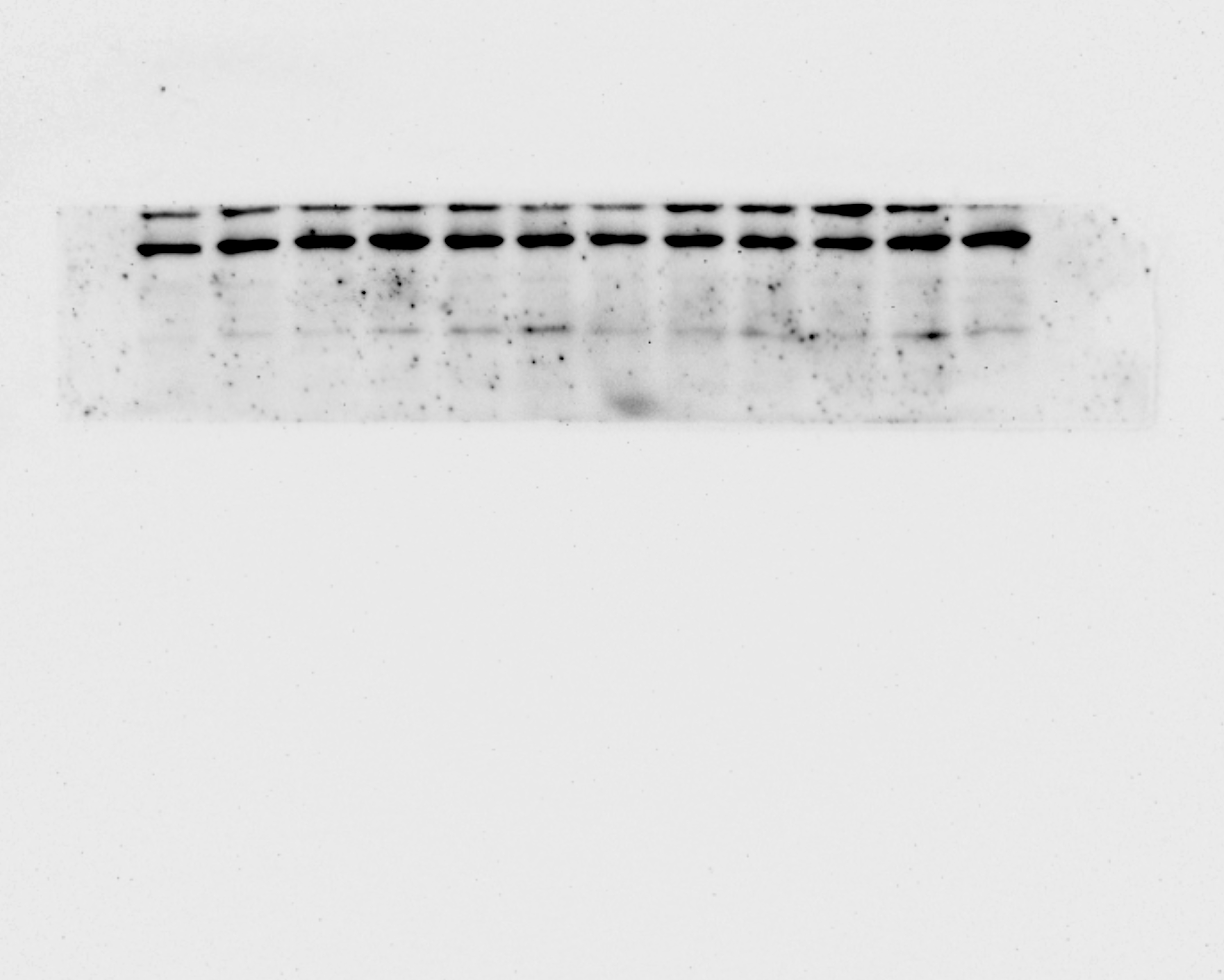


47 kDa

Caspase-9

Note: the exposure time of protein bands is 36s.

**
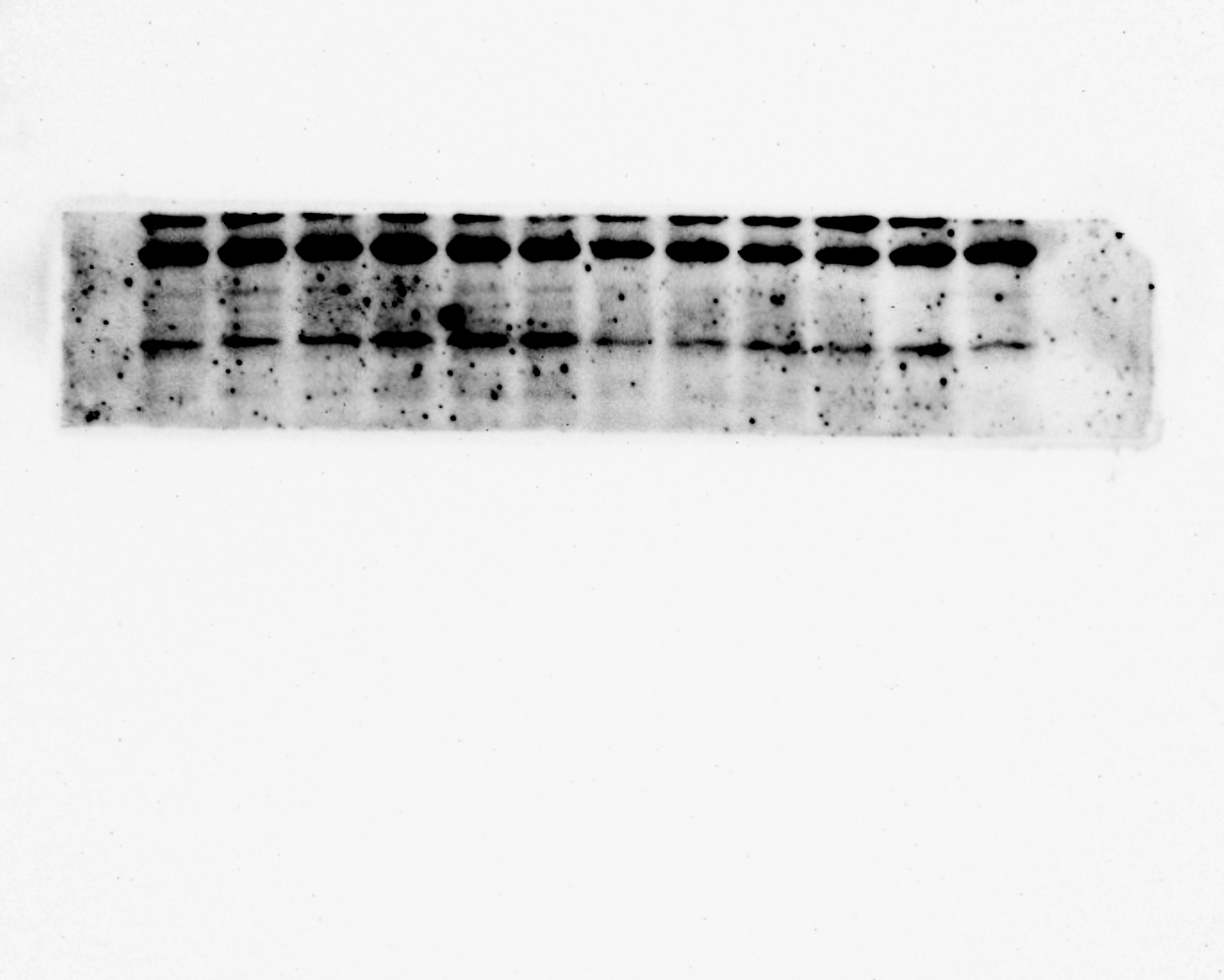
**

35 kDa

47 kDa

Cleaved-Caspase-9

Caspase-9

Note: the exposure time of protein bands is 60s.

**Figure 6G**


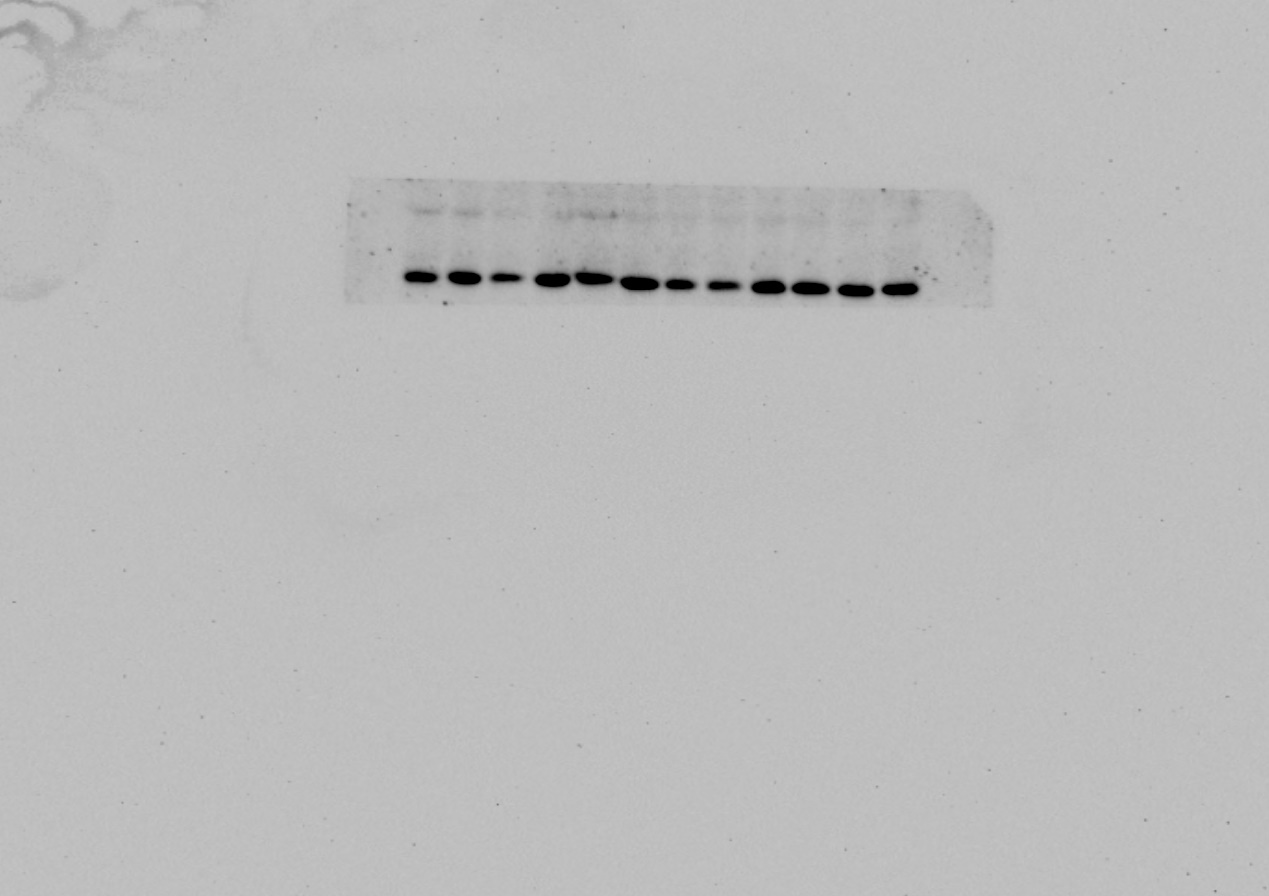


35 kDa

Caspase-3


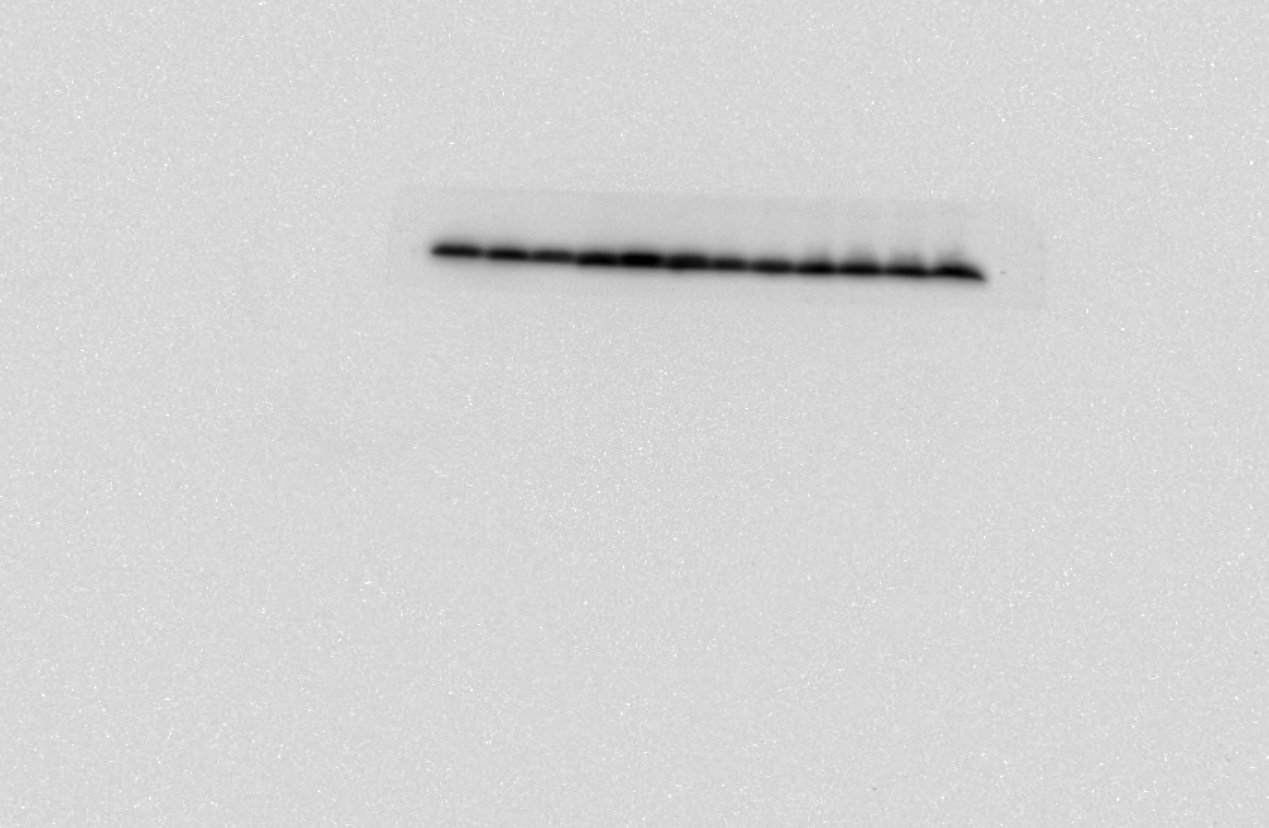


17 kDa

Cleaved-Caspase-3

**Figure 6G**


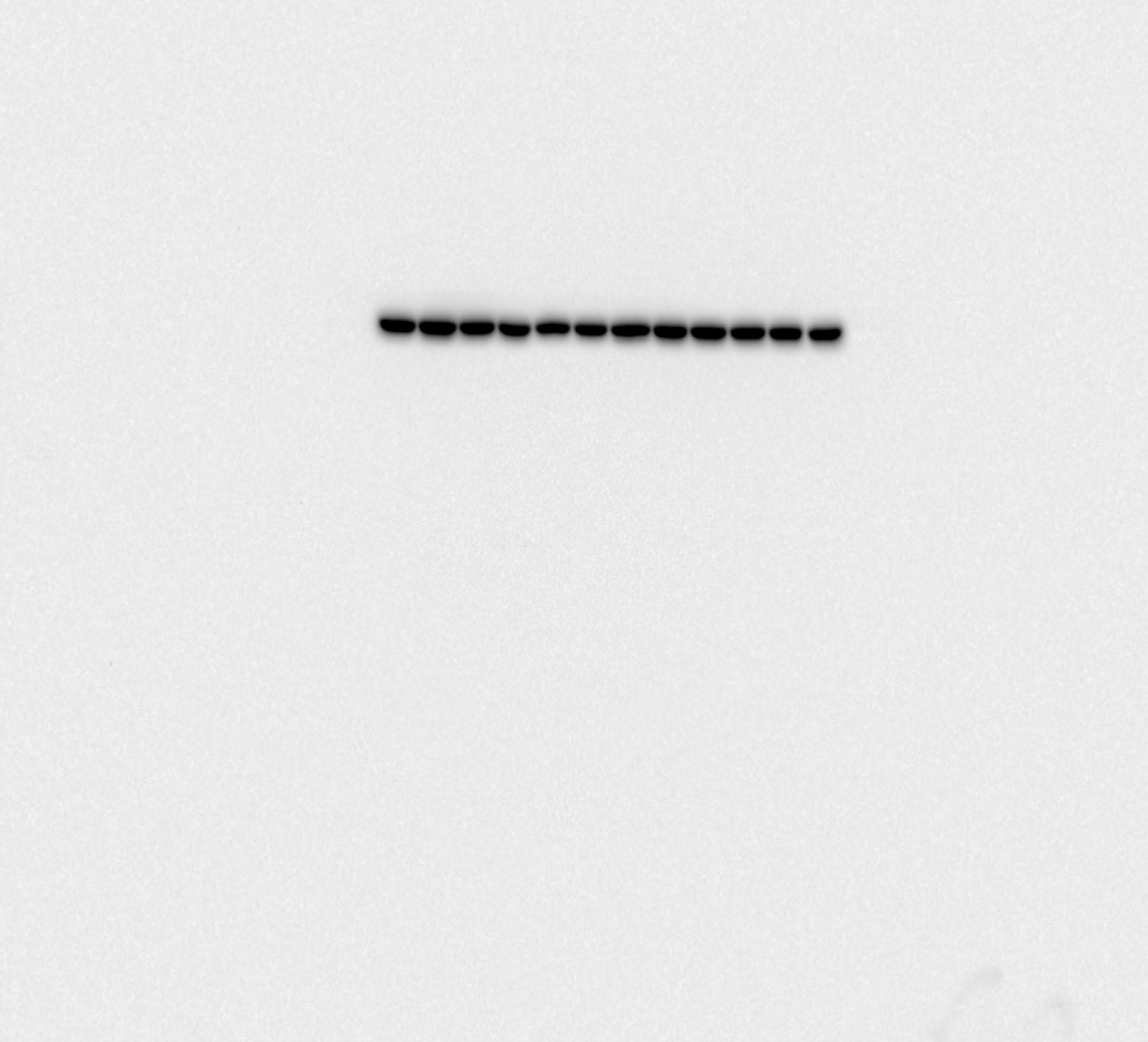


43 kDa

β-actin

**Figure 7A**


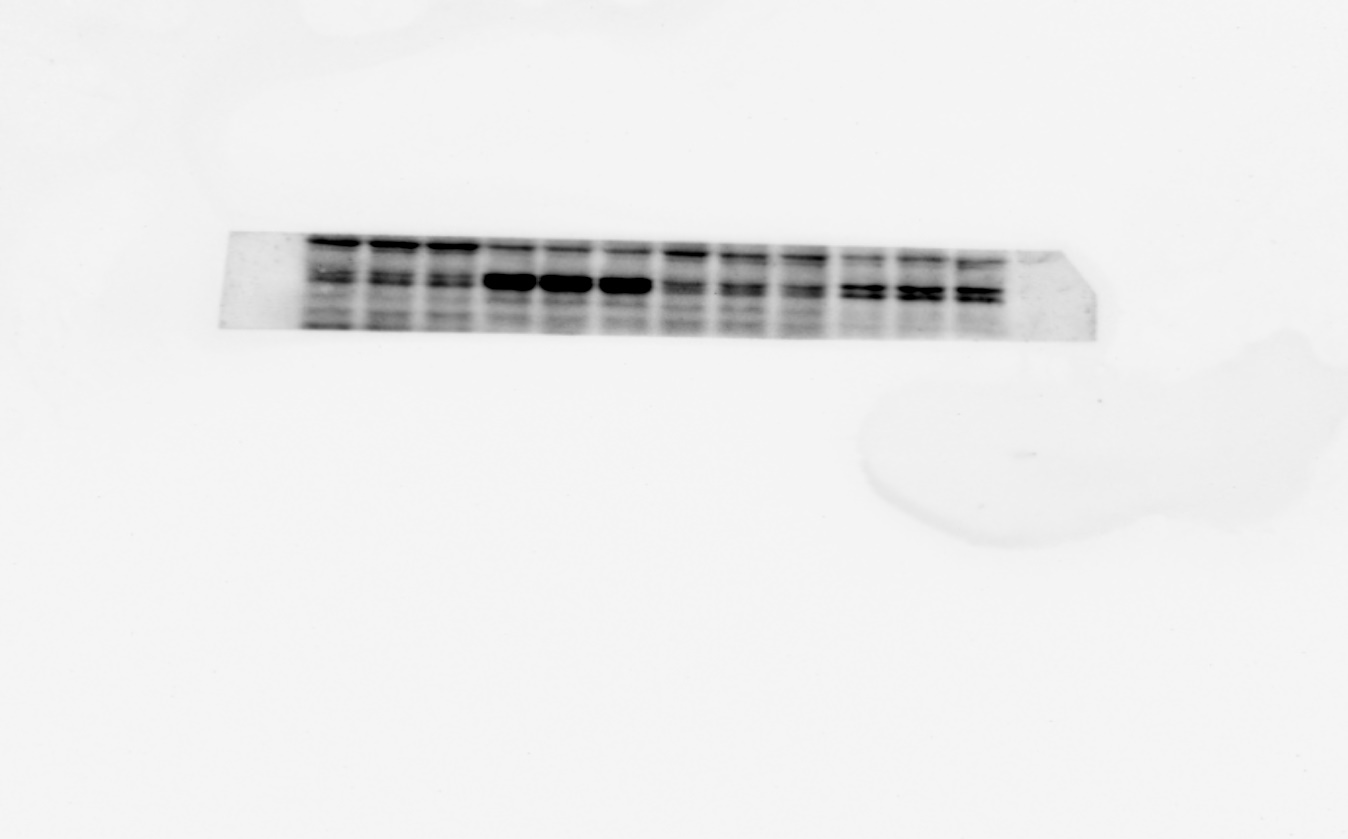


41 kDa

p-P_38_MAPK


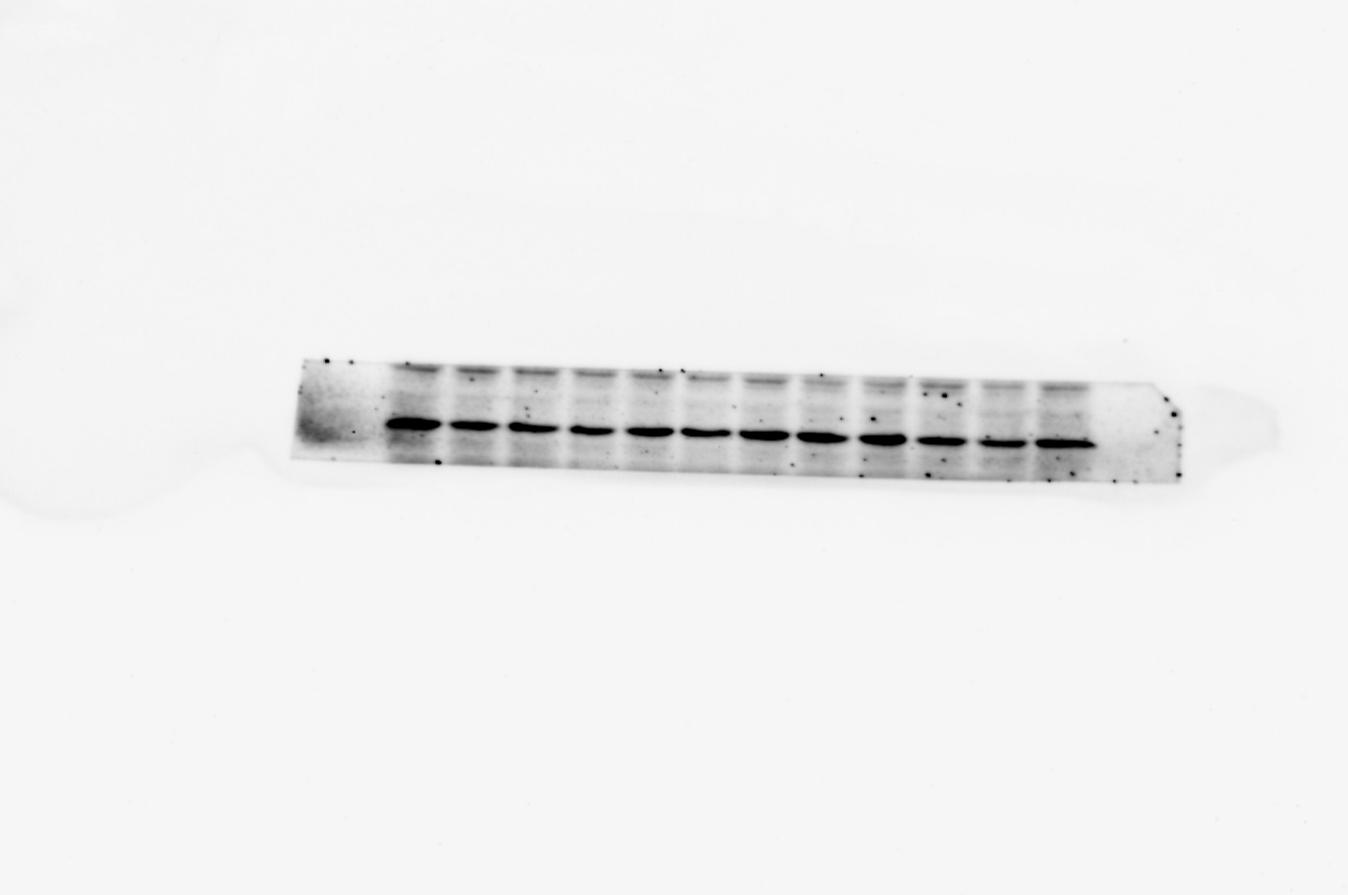


41 kDa

P_38_MAPK

**Figure 7A**


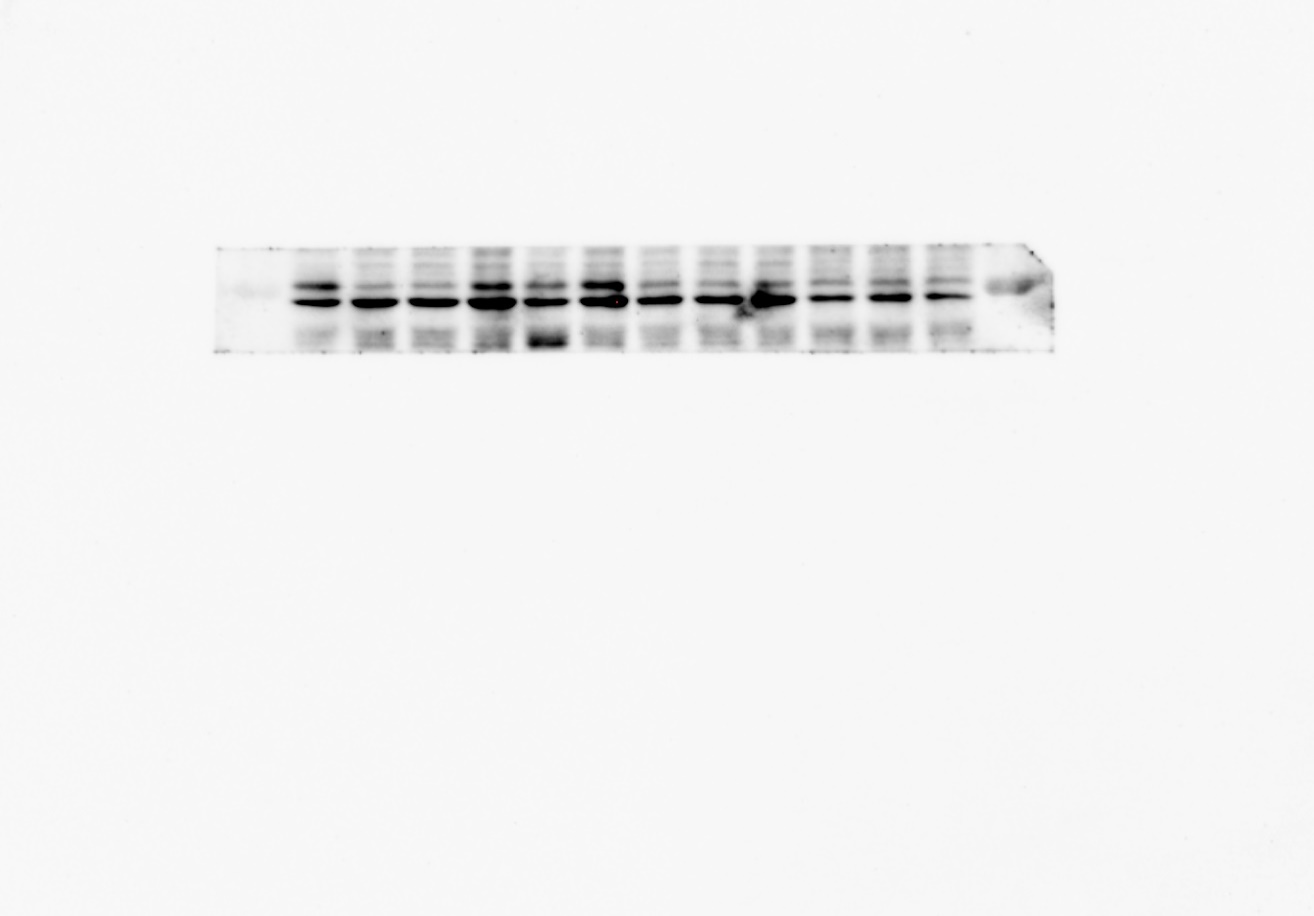


46 kDa

54 kDa

p-JNK


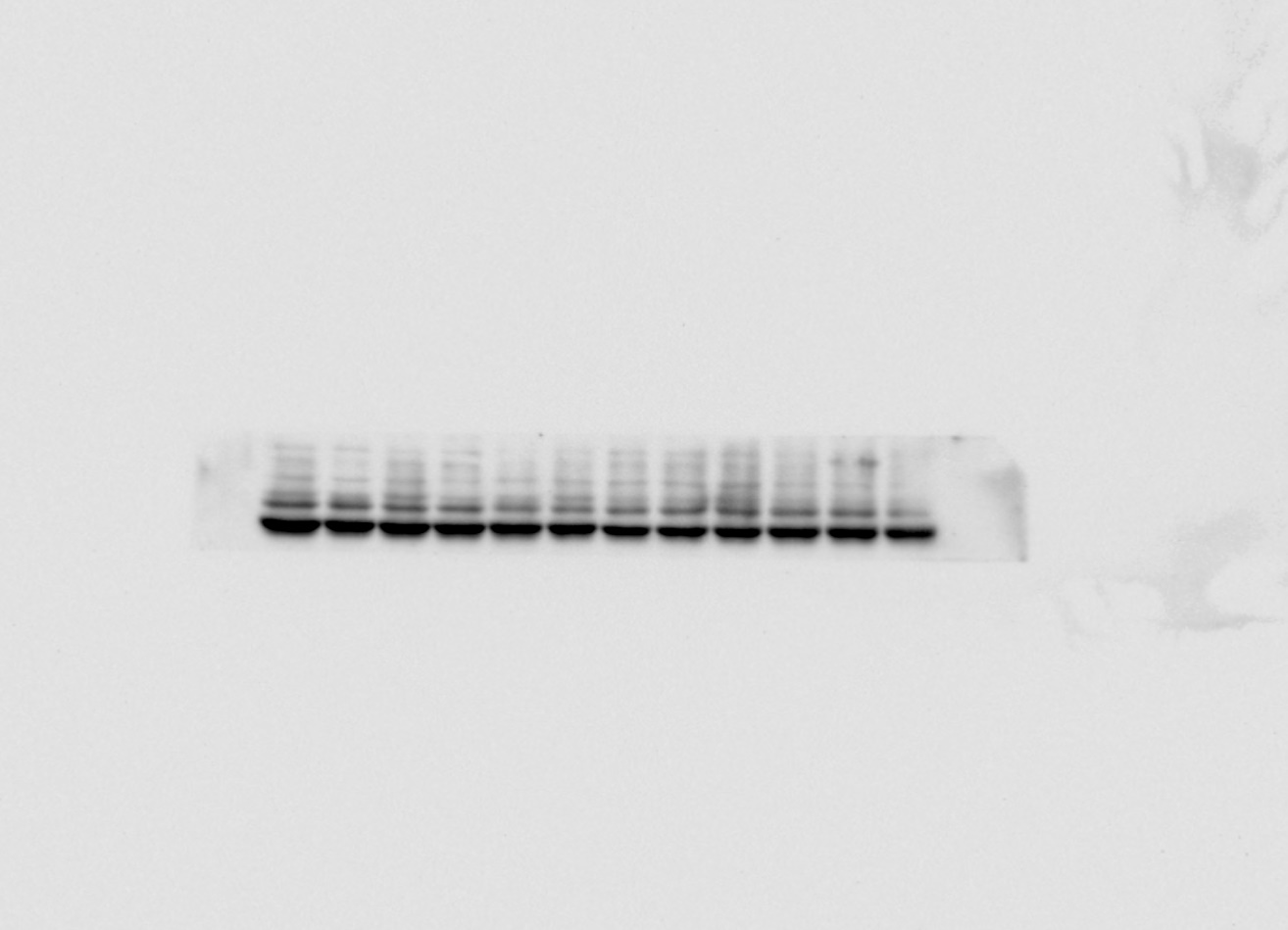


46 kDa

54 kDa

JNK

**Figure 7A**


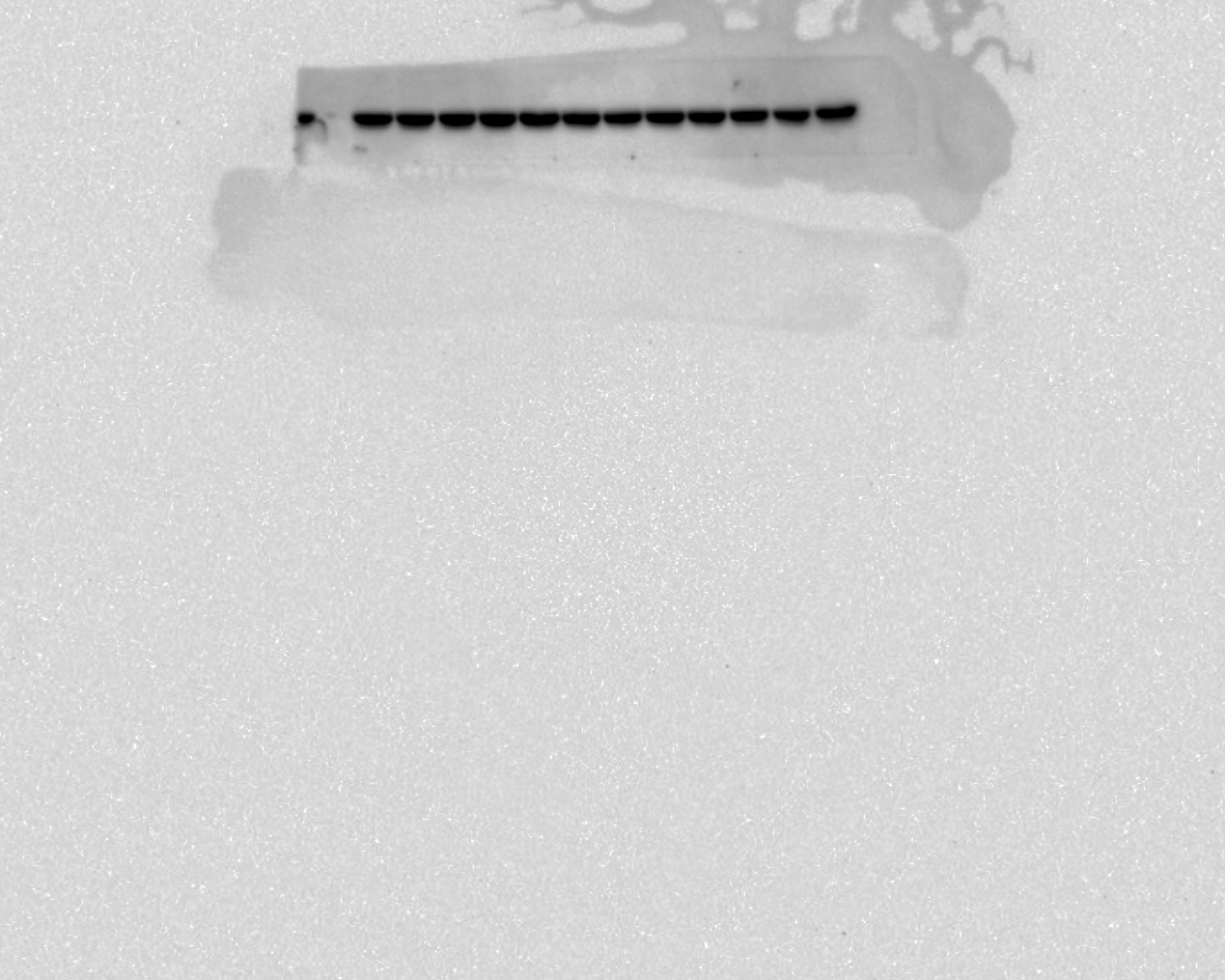


43 kDa

β-actin

**Figure 8A**


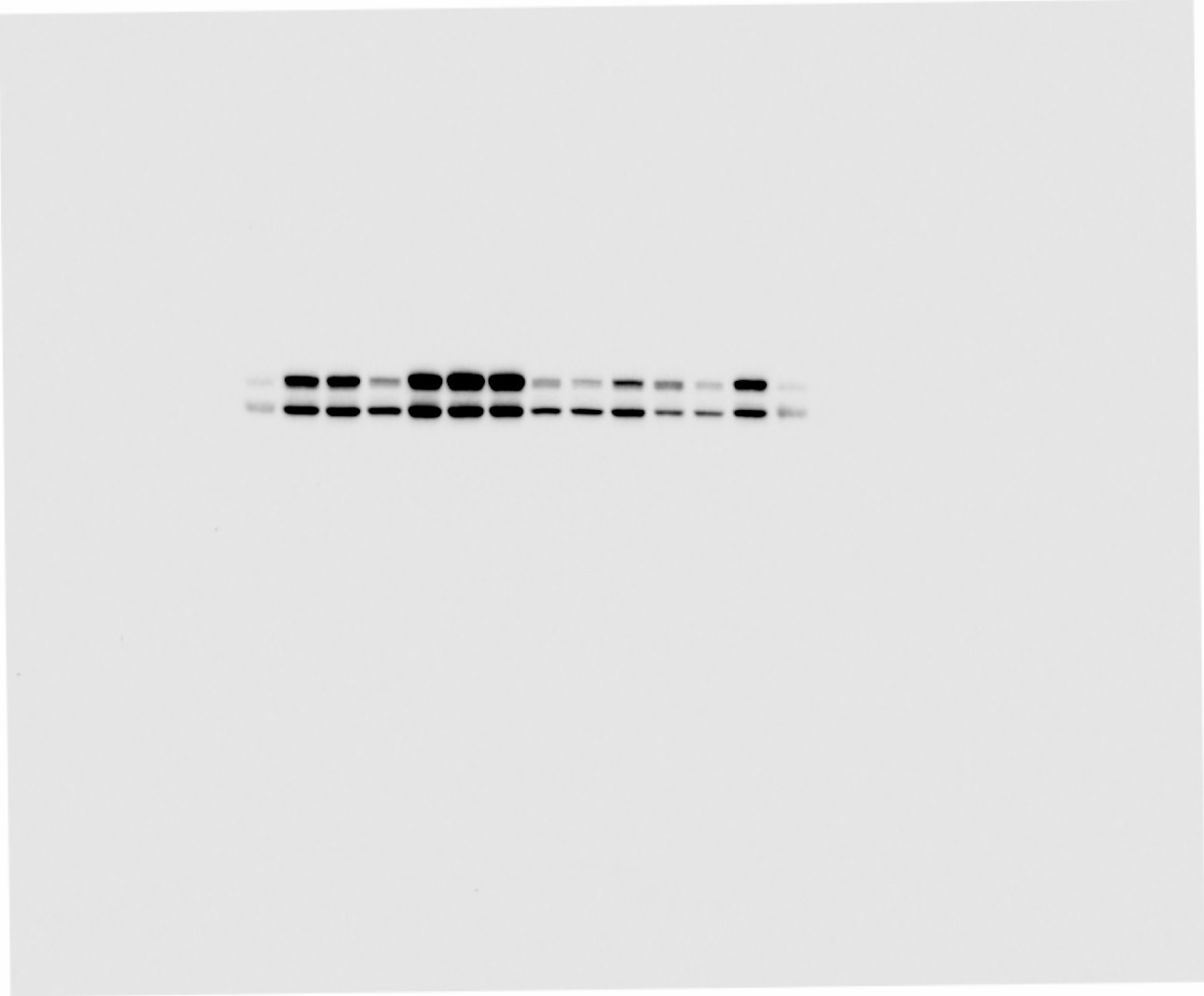


78 kDa

GRP78


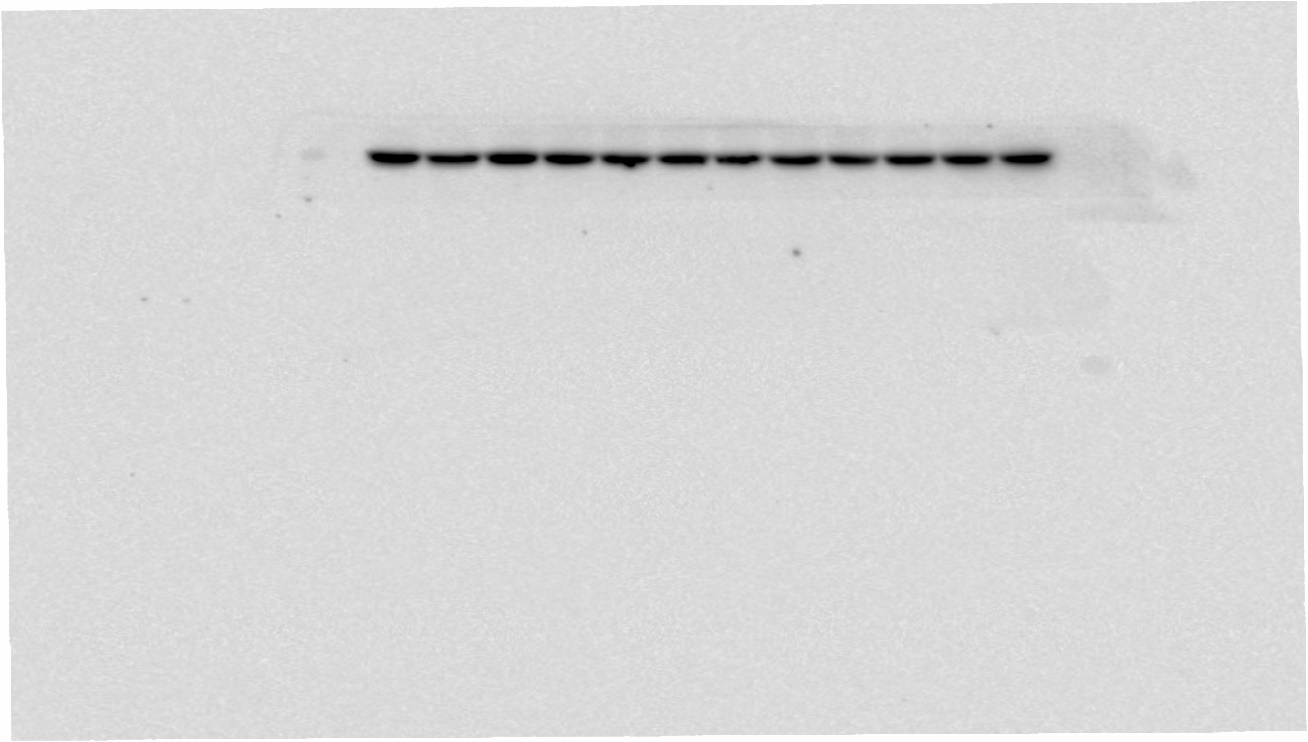


43 kDa

β-actin

**Figure 8C**


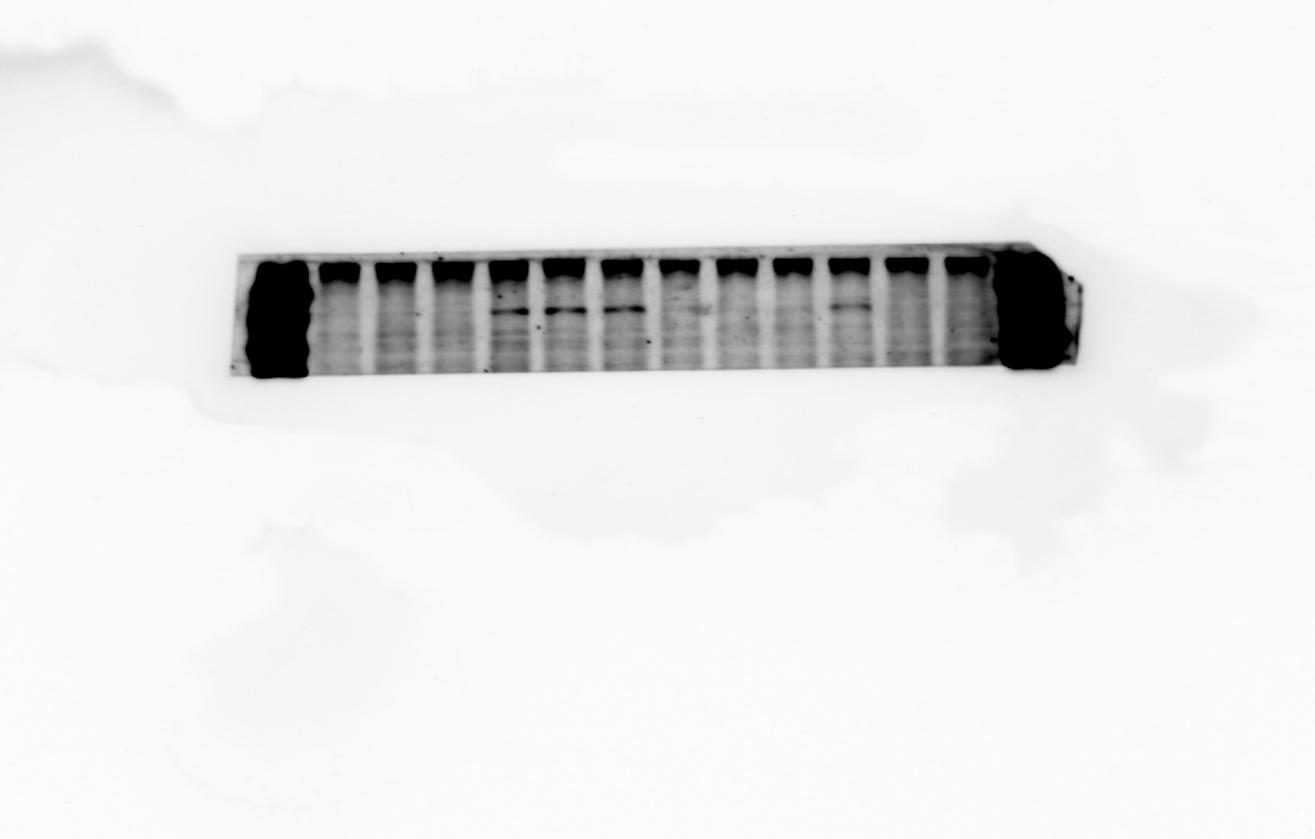


140 kDa

p-PERK


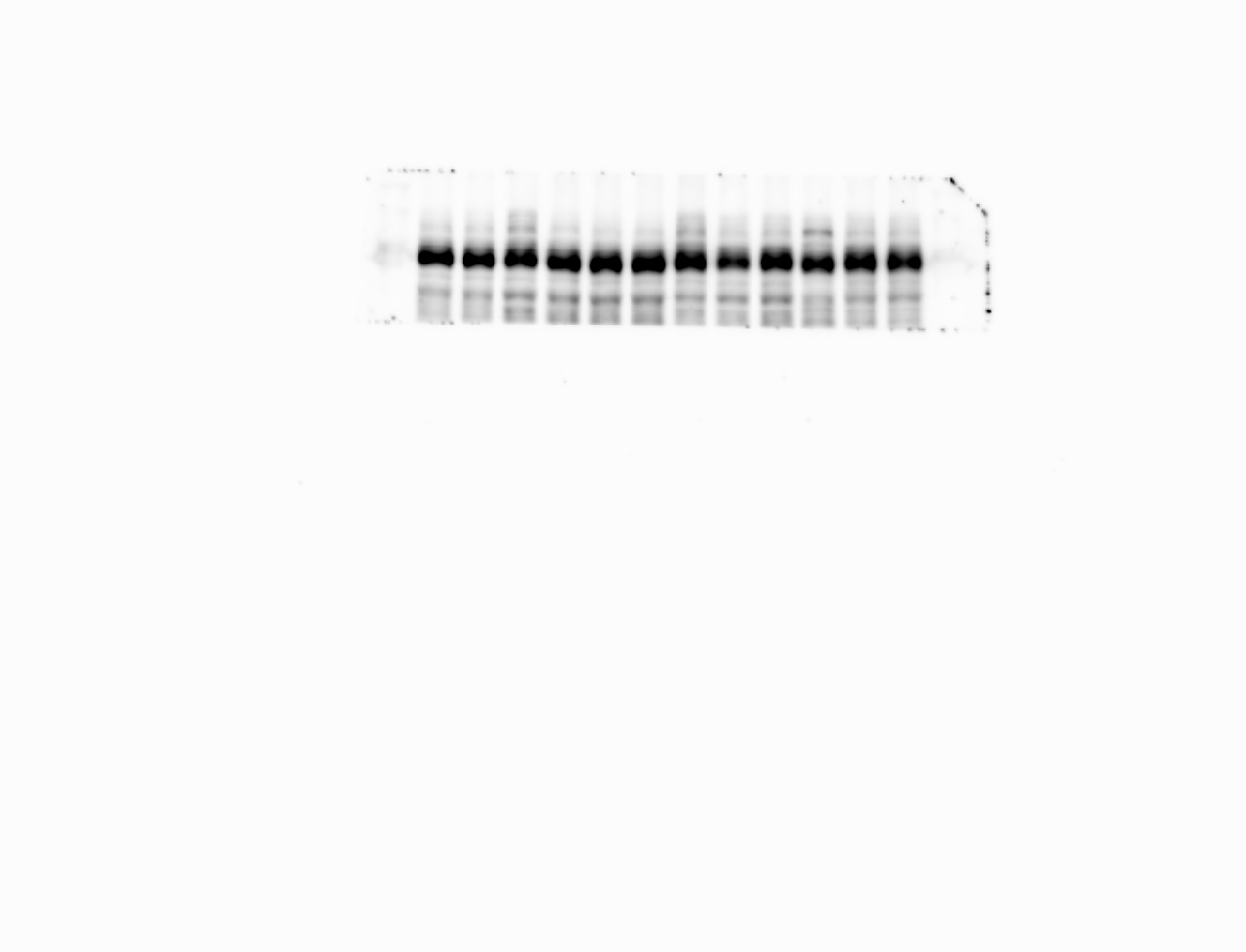


140 kDa

PERK

**Figure 8C**


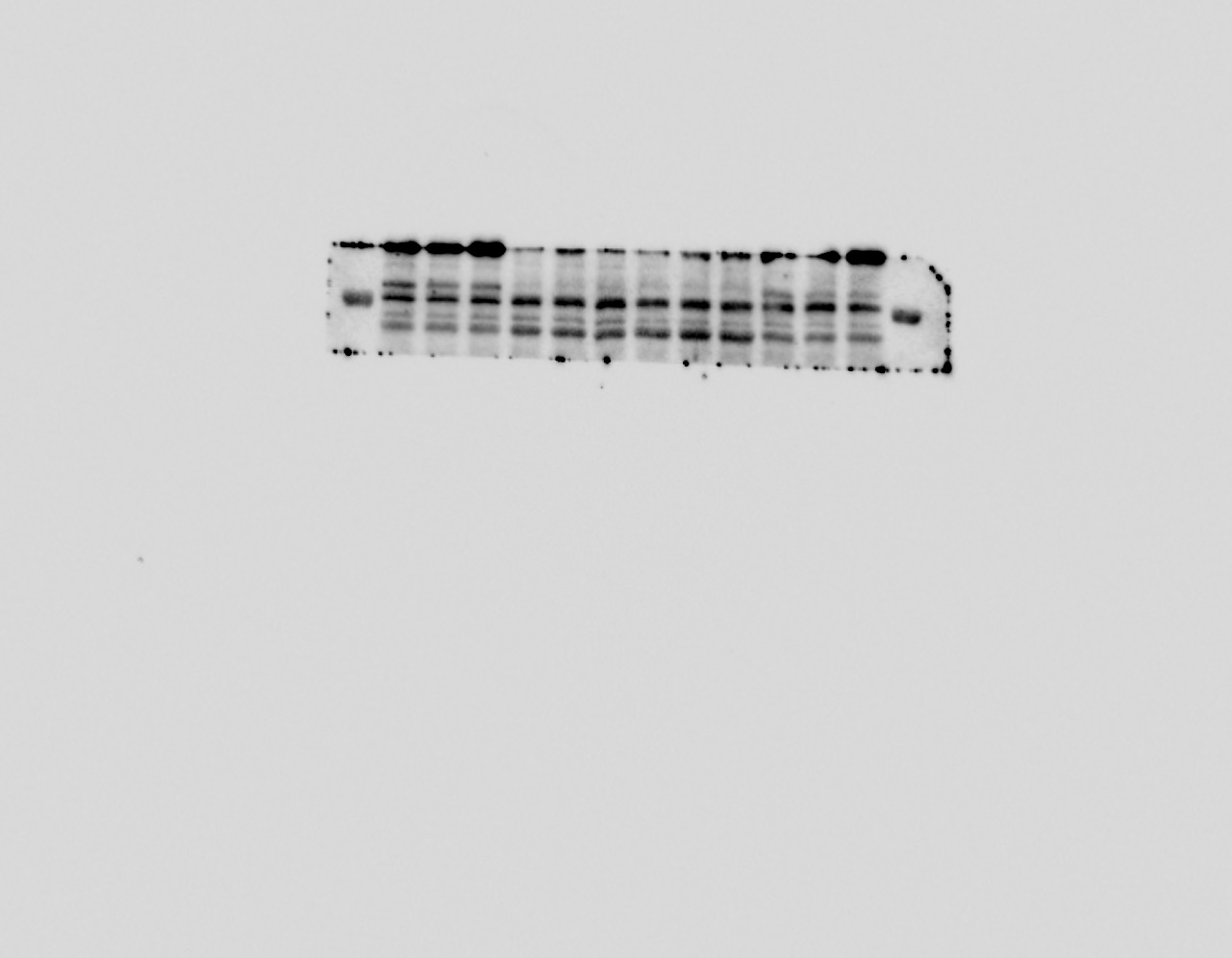


110 kDa

p-IRE1


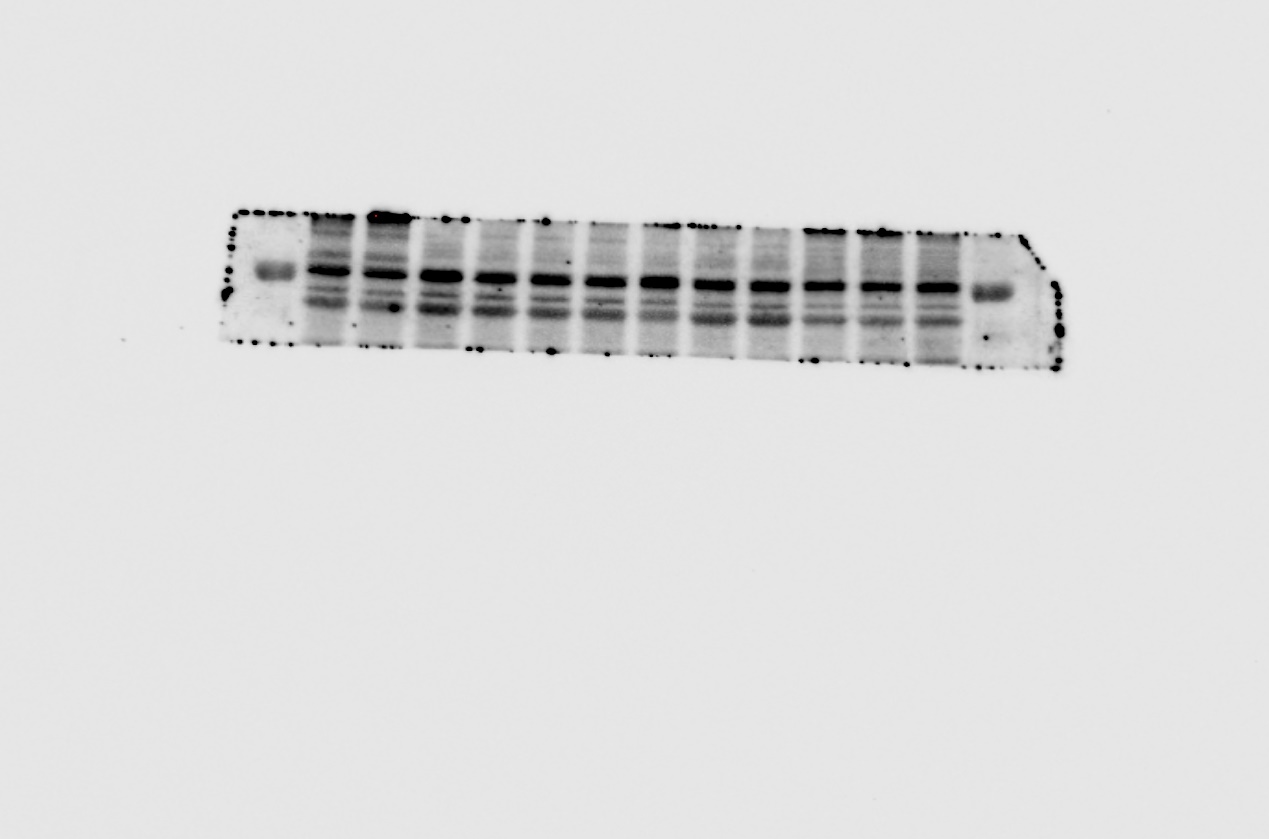


110 kDa

IRE1

**Figure 8C**


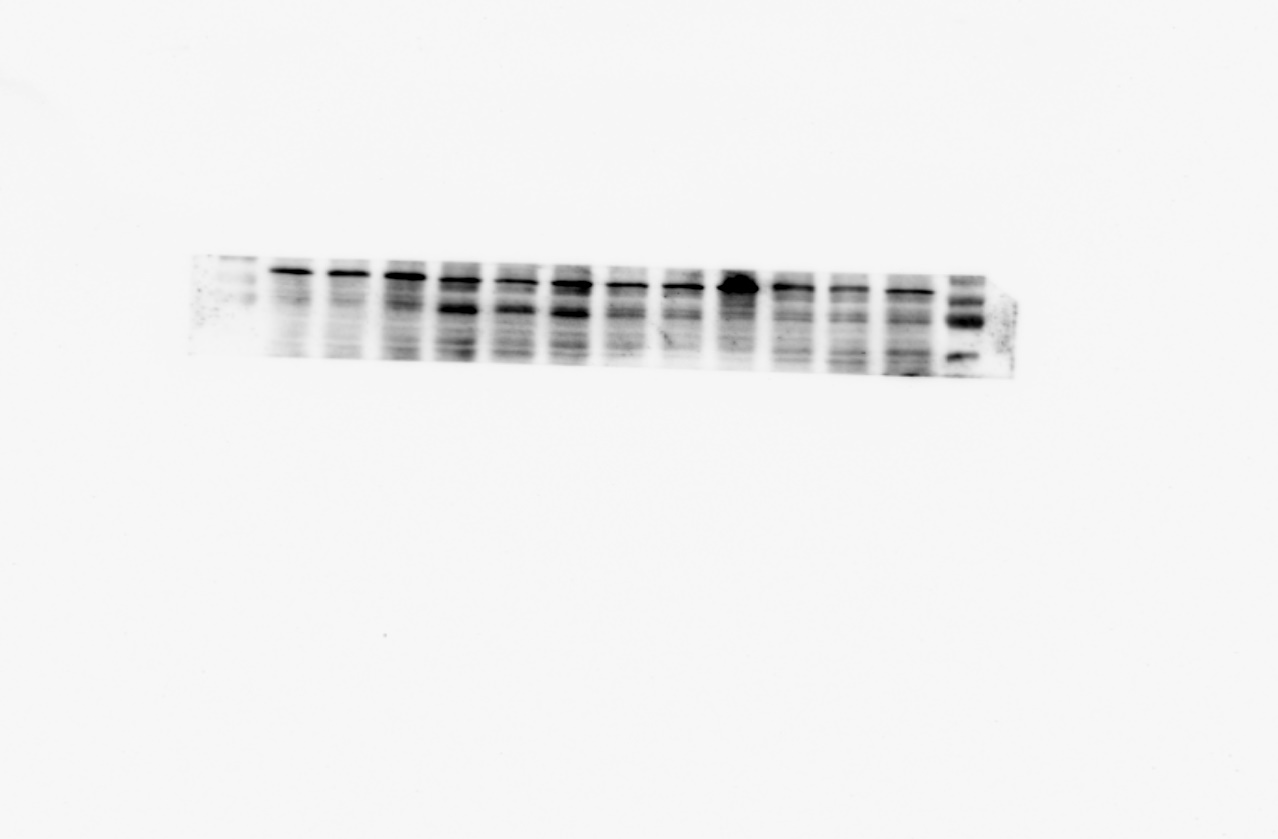


75 kDa

ATF6


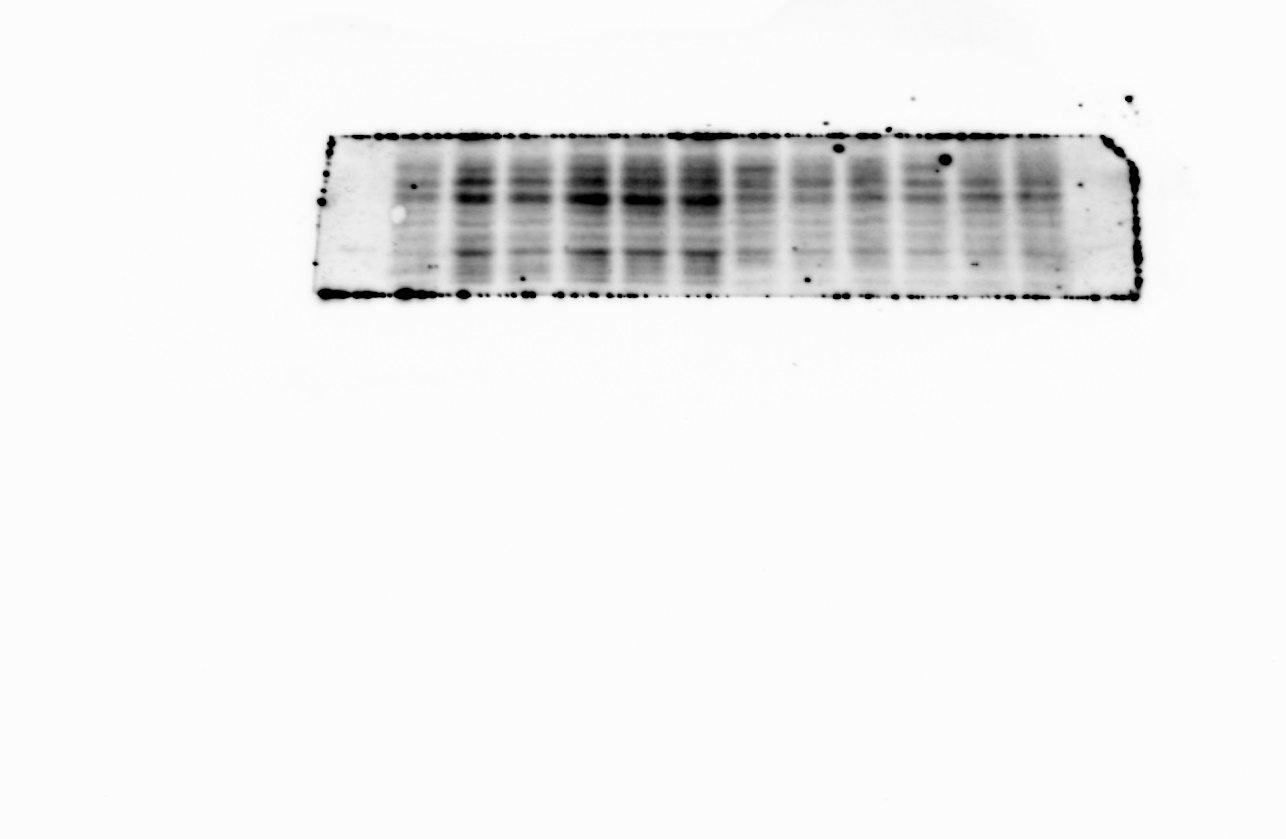


27 kDa

CHOP

**Figure 8C**


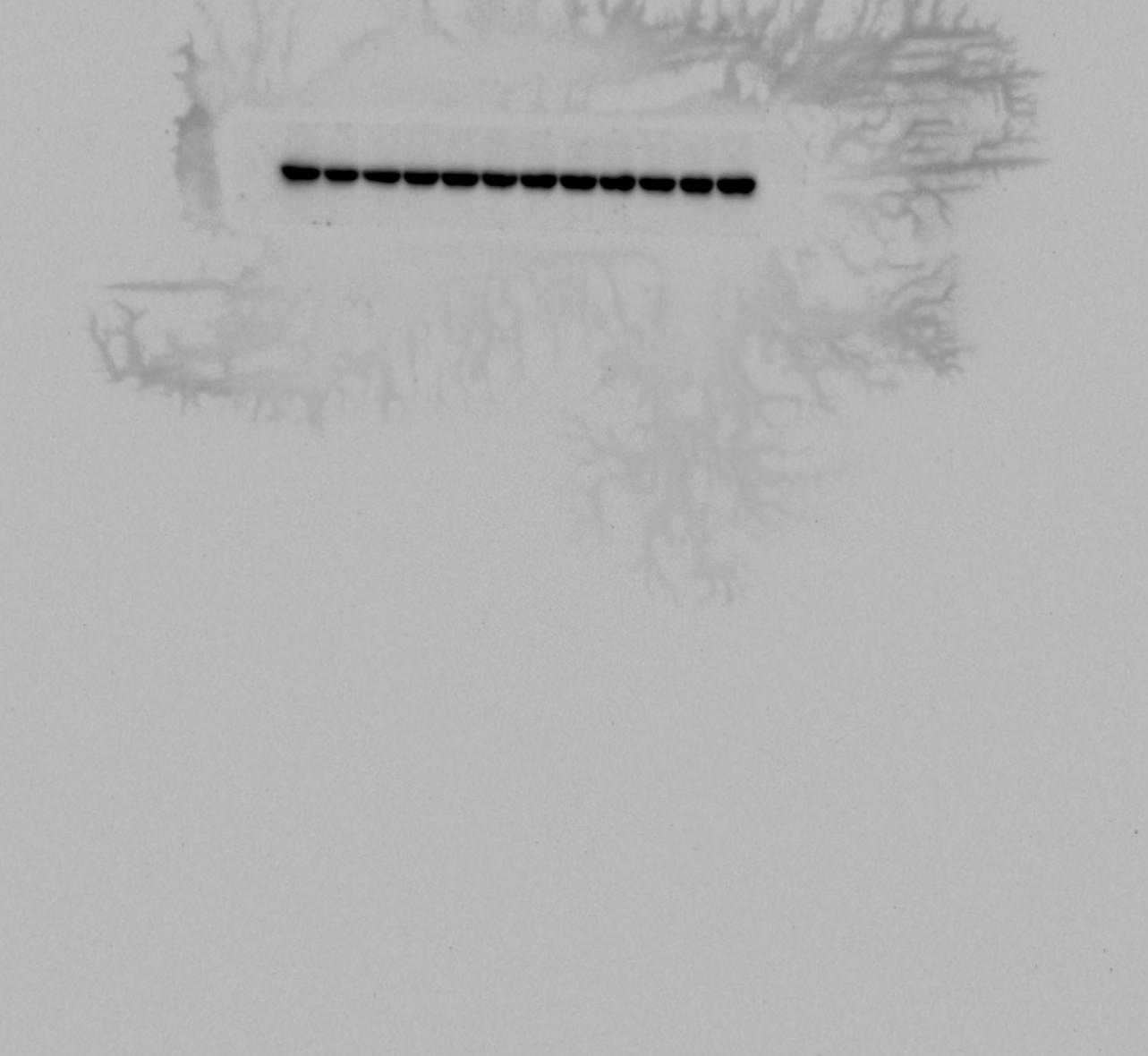


43 kDa

β-actin
